# Glucocorticoid regulation of ATP release from spinal astrocytes underlies diurnal exacerbation of neuropathic mechanical allodynia

**DOI:** 10.1038/ncomms13102

**Published:** 2016-10-14

**Authors:** Satoru Koyanagi, Naoki Kusunose, Marie Taniguchi, Takahiro Akamine, Yuki Kanado, Yui Ozono, Takahiro Masuda, Yuta Kohro, Naoya Matsunaga, Makoto Tsuda, Michael W. Salter, Kazuhide Inoue, Shigehiro Ohdo

**Affiliations:** 1 Department of Pharmaceutics, Faculty of Pharmaceutical Sciences, Kyushu University, 3-1-1 Maidashi, Higashi-ku, Fukuoka 812-8582, Japan; 2 Department of Glocal Healthcare, Faculty of Pharmaceutical Sciences, Kyushu University, 3-1-1 Maidashi, Higashi-ku, Fukuoka 812-8582, Japan; 3 Department of Molecular and System Pharmacology, Faculty of Pharmaceutical Sciences, Kyushu University, 3-1-1 Maidashi, Higashi-ku, Fukuoka 812-8582, Japan; 4 Department of Life Innovation, Faculty of Pharmaceutical Sciences, Kyushu University, 3-1-1 Maidashi, Higashi-ku, Fukuoka 812-8582, Japan; 5 Program in Neuroscience and Mental Health, Hospital for Sick Children, Toronto, Ontario, Canada M5G 1X8; 6 Department of Physiology, University of Toronto, Toronto, Ontario, Canada M5S 1A8; 7 University of Toronto Centre for the Study of Pain, Toronto, Ontario, Canada M5T 1P8

## Abstract

Diurnal variations in pain hypersensitivity are common in chronic pain disorders, but the underlying mechanisms are enigmatic. Here, we report that mechanical pain hypersensitivity in sciatic nerve-injured mice shows pronounced diurnal alterations, which critically depend on diurnal variations in glucocorticoids from the adrenal glands. Diurnal enhancement of pain hypersensitivity is mediated by glucocorticoid-induced enhancement of the extracellular release of ATP in the spinal cord, which stimulates purinergic receptors on microglia in the dorsal horn. We identify serum- and glucocorticoid-inducible kinase-1 (SGK-1) as the key molecule responsible for the glucocorticoid-enhanced release of ATP from astrocytes. SGK-1 protein levels in spinal astrocytes are increased in response to glucocorticoid stimuli and enhanced ATP release by opening the pannexin-1 hemichannels. Our findings reveal an unappreciated circadian machinery affecting pain hypersensitivity caused by peripheral nerve injury, thus opening up novel approaches to the management of chronic pain.

## Abstract (web summary)

Neuropathic pain hypersensitivity is known to undergo diurnal variations, although the underlying mechanisms are not clear. Using a sciatic nerve-injury mouse model, the authors find such diurnal changes are mediated by glucocorticoid induced enhancement of ATP release from astrocytes via pannexin-1 hemichannels.

## Graphical abstract

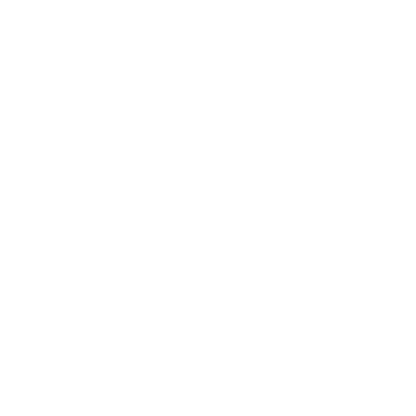

A variety of pathological conditions exhibit profound day–night changes in the symptom intensity with a large portion exacerbating and occurrence of grave events. Diurnal alterations in pain hypersensitivity have been confirmed in patients with cancer^1^,^2^, rheumatoid arthritis^3^, diabetic neuropathy^4^, fibromyalgia^5^ and multiple sclerosis^6^; however, the underlying mechanism remains unknown.

Pain hypersensitivity is often caused by peripheral nerve injury, which is associated with the hyperexcitability of neurons in the dorsal horn of the spinal cord^7^,^8^,^9^. Extracellular ATP in the spinal cord has been implicated in the development and maintenance of pain hypersensitivity after nerve injury^10^,^11^,^12^,^13^,^14^. This pain hypersensitivity is even evoked by a simple touch, pressure from clothing or gentle massage, which is also known as ‘mechanical allodynia'^15^,^16^.

In mammals, diurnal rhythms in physiological functions are governed by an internal self-sustained molecular oscillator referred to as the circadian clock^16^,^17^. The circadian clock system in mammals is composed of a master pacemaker, which is located in the suprachiasmatic nuclei (SCN) of the anterior hypothalamus, and subsidiary oscillators in other brain regions, as well as many peripheral tissues^18^,^19^. The times of the day-dependent changes in the secretion of glucocorticoids from the adrenal glands are controlled by the SCN^20^, which, in turn, synchronizes subsidiary oscillators to coordinate various biological processes, thereby generating daily rhythms in physiology and behaviour^21^,^22^.

Since the glucocorticoid receptor (GCR) is expressed in most cell types, including spinal neurons, microglia and astrocytes^23^,^24^,^25^, we investigated whether the diurnal secretion of adrenal glucocorticoids affects the threshold of mechanical allodynia in peripheral nerve-injured mice. Temporal elevations in glucocorticoid levels enhance the extracellular release of ATP in the spinal cord, which stimulates purinergic receptors on microglia in the dorsal horn. The stimuli of purinergic receptors decrease the threshold of mechanical allodynia. Serum- and glucocorticoid-inducible kinase-1 (SGK-1) mediates the glucocorticoid-enhanced release of ATP from astrocytes by opening the pannexin-1 hemichannels. Therefore, our findings reveal an underlying mechanism of diurnal exacerbation of neuropathic mechanical allodynia and also provide novel approaches to the management of chronic pain.

## Results

### Glucocorticoids govern diurnal exacerbation of mechanical allodynia

**Figure 1:**
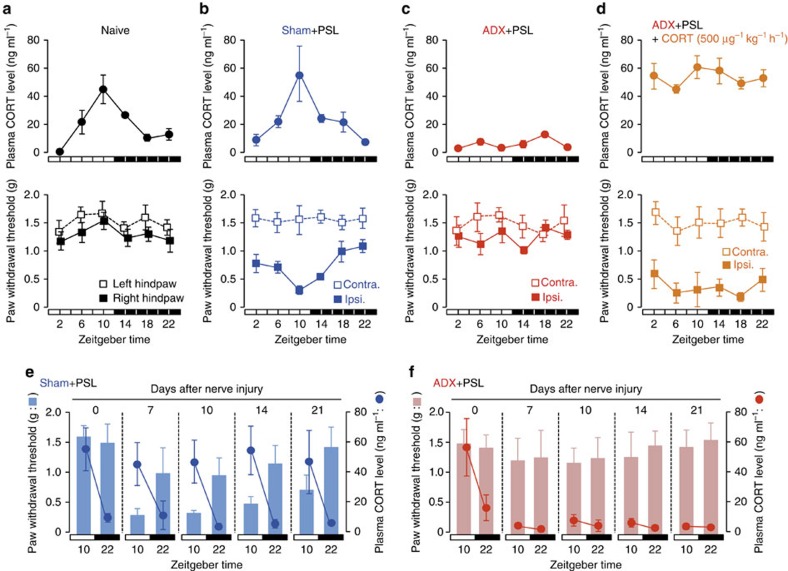
Glucocorticoids regulate the diurnal oscillations in the threshold of mechanical allodynia in PSL mice. (**a**–**d**) Temporal profiles of plasma glucocorticoid levels and the paw withdrawal threshold in naive (**a**), sham+PSL (**b**), ADX+PSL (**c**) and CORT (500 μg kg^−1^ h^−1^)-administered ADX+PSL (**d**) male mice (means±s.e.m.; *n*=5–6). The continuous administration of CORT (500 μg kg^−1^ h^−1^) to ADX+PSL mice was performed by the subcutaneous (s.c.) implantation of an osmotic minipump. The withdrawal threshold of the nerve-injured ipsilateral (ipsi.) right hindpaw and sham-operated contralateral (contra.) left hindpaw of sham+PSL, ADX+PSL, and CORT (500 μg kg^−1^ h^−1^)-administered ADX+PSL mice was assessed by the von Frey up-down method. The withdrawal threshold of the right and left hindpaws of naive mice was also assessed by the same method. Significant time-dependent variations are observed in plasma CORT levels in naive (*F*_5,30_=13.128, *P*<0.001; ANOVA) and sham+PSL (*F*_5,30_=6.592, *P*<0.001; ANOVA) mice, and in the withdrawal threshold of the ipsilateral hindpaw of sham+PSL (*F*_5,30_=5.482, *P*=0.011; ANOVA) mice. (**e**,**f**), An inverse relationship between diurnal oscillations in plasma CORT levels and the paw withdrawal threshold in sham+PSL (**e**) and ADX+PSL (**f**) male mice after nerve injury (means±s.e.m.; *n*=6). Significant time-dependent variations are observed in plasma CORT levels (*F*_7,40_=3.359, *P*=0.008; ANOVA) and in the withdrawal threshold of the ipsilateral hindpaw in sham+PSL (*F*_7,40_=2.655, *P*=0.024; ANOVA) mice. Neither plasma CORT levels (*F*_7,40_=0.926, *P*=0.497; ANOVA) nor the withdrawal threshold of the ipsilateral hindpaw (*F*_7,40_=0.647, *P*=0.715; ANOVA) show significant time-dependent variations in ADX+PSL mice.

To determine the pathological relevance of the diurnal secretion of adrenal glucocorticoids in neuropathic hypersensitivity, we investigated the influence of adrenalectomy on the threshold of mechanical allodynia in nerve-injured mice. To achieve this, we prepared adrenalectomized (ADX) and sham-operated (sham) male ICR mice (Fig. 1). To induce neuropathic pain hypersensitivity, ADX mice underwent partial sciatic nerve ligation (PSL) in the right hindlimb. PSL is a well-established nerve injury model which produces pain hypersensitivity lasting >3 weeks^26^. All animals were maintained on a 12-h light–dark cycle (ZT, zeitgeber time; ZT0, lights on; ZT12, lights off). Plasma CORT (corticosterone) concentrations in animals that had not been subjected to nerve injury—naive mice—showed significant diurnal oscillations (*F*_5,30_=13.128, *P*<0.001; Fig. 1a, upper panel). The CORT levels increased from the late light phase to the early dark phase. A significant diurnal oscillation in plasma CORT levels was also observed in male sham+PSL mice on day 7 after nerve injury (*F*_5,30_=6.592, *P*<0.001; Fig. 1b, upper panel). No significant differences were observed in the rhythmicity of plasma CORT levels between sham+PSL and naive male mice.

We found that the withdrawal threshold of the ipsilateral hindpaw, but not that of the contralateral hindpaw, of male sham+PSL mice exhibited a significant diurnal oscillation (*F*_5,30_=5.482, *P*=0.011; Fig. 1b, lower panel). By contrast, no diurnal variation was noted in the paw withdrawal threshold in male naive mice (Fig. 1a, lower panel). The lowest level of paw withdrawal, that is, maximum pain hypersensitivity, in male sham+PSL mice occurred from the late light phase to the early dark phase (Fig. 1b, lower panel). The trough level of paw withdrawal corresponded to the peak in plasma CORT levels in male sham+PSL mice. This inverse relationship was observed between the threshold of mechanical allodynia and plasma CORT levels, for the entire 3-week observation period after PSL (Fig. 1e).

In contrast to naive and sham+PSL mice, there was no diurnal oscillation in plasma CORT levels male ADX+PSL mice (*F*_5,24_=1.271, *P*=0.309; Fig. 1c, upper panel); CORT levels remained low throughout the day. Furthermore, the withdrawal threshold of the ipsilateral hindpaw in male ADX+PSL mice did not decrease even after nerve injury (*F*_5,24_=1.131, *P*=0.371; Fig. 1c, lower panel), which was similar to the paw withdrawal threshold in the contralateral side. Although we continued to assess the paw withdrawal threshold of male ADX+PSL mice for 3 weeks after nerve injury, no decreases in the pain threshold were observed during the duration of the experimental period (Fig. 1f).

To determine whether pain hypersensitivity could be reproduced in male ADX+PSL mice, we administered CORT (500 μg kg^−1^ h^−1^, s.c.) to animals starting 3 days after PSL. We found that the withdrawal threshold of the ipsilateral paw decreased to a level that was not different from the minimum level in sham+PSL mice (Fig. 1d). By contrast, the administration of CORT had no effect on the withdrawal threshold in the contralateral paw. In these experiments, CORT was administered continuously and neither plasma CORT levels nor the withdrawal threshold of the ipsilateral hindpaw in CORT-administered male ADX+PSL mice showed a significant diurnal oscillation (*F*_5,24_=1.073, *P*=0.408 for CORT levels, *F*_5,24_=0.591, *P*=0.707 for pain threshold; Fig. 1d). We also detected significant diurnal variations in the threshold of pain hypersensitivity of female sham+PSL mice (Supplementary Fig. 1). Consistent with males, diurnal variations in pain intensity was dampened by adrenalectomy. Therefore, the decrease observed in the threshold of pain hypersensitivity in PSL mice and the diurnal variations in the hypersensitivity appeared to be dependent on diurnal oscillations in glucocorticoid levels.

**Figure 2:**
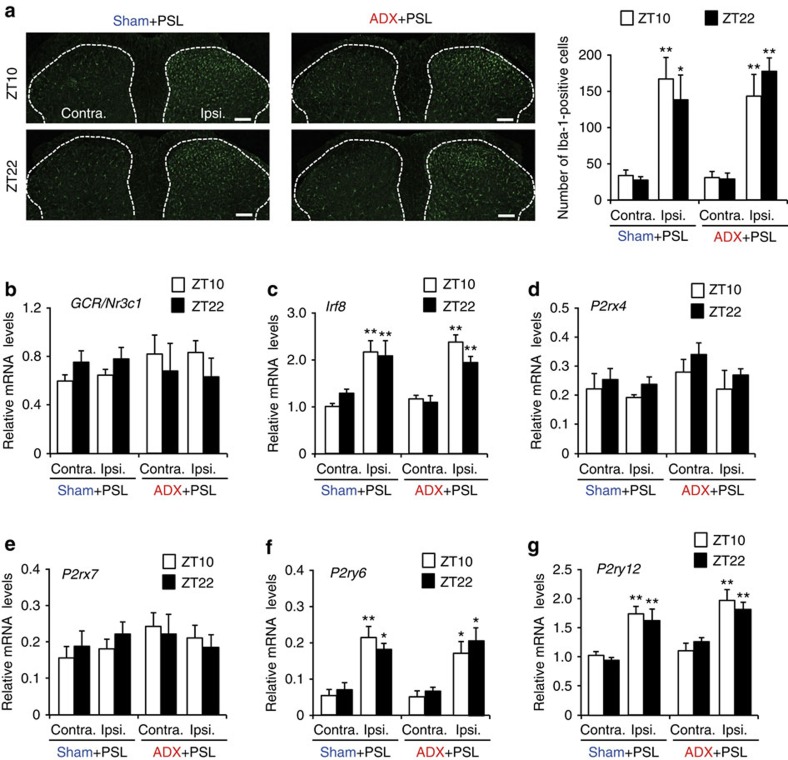
Morphological and molecular alterations in the spinal cords of PSL mice. (**a**) Temporal profiles of Iba1-positive cells in the spinal cords of sham+PSL and ADX+PSL male mice. The dorsal horn areas are surrounded with dashed line. Scale bar, 100 μm. The right panel shows the quantification of the number of Iba1-positive cells (means±s.e.m.; *n*=5). Tukey–Kramer's *post hoc* tests (PHT) shows that the number of Iba1-positive cells in the ipsilateral (Ipsi.) side of sham+PSL and ADX+PSL mice is significantly higher than that in the contralateral (Contra.) side (**P<0.01, *P<0.05), whereas no significant difference is noted in the number of cells between the two time points (*F*_7,32_=6.550, *P*<0.001; ANOVA). (**b**–**g**) Temporal profiles of mRNA levels for GCR/*Nr3c1*, *Irf8*, *P2rx4*, *P2rx7*, *P2ry6* and *P2ry12* in the spinal cords of sham+PSL and male ADX+PSL mice (means±s.e.m.; *n*=5–6). **P<0.01, *P<0.05 significantly different from contralateral sides in the corresponding groups (*F*_7,36_=13.307, *P*<0.001 for *Irf8*, *F*_7,36_=7.586, *P*<0.001 for *P2ry6*, *F*_7,36_=9.544, *P*<0.001 for *P2ry12*; ANOVA with Tukey–Kramer's PHT).

Since purinergic receptors on microglia in the spinal cord are responsible for the induction of pain hypersensitivity evoked by a peripheral nerve injury^12^,^13^, the stimulation of spinal microglia by ATP is required for the development and maintenance of neuropathic pain hypersensitivity. Although an increase in the number of Iba1-positive cells, a marker of microglia, was observed in the ipsilateral side of the spinal cord in male sham+PSL mice (*F*_7,32_=6.550, *P*<0.001; Fig. 2a), no significant time-dependent changes were noted in the number of Iba1-positive cells. We also detected elevations in the mRNA levels of *interferon regulatory factor 8* (*Irf8*; *F*_7,36_=13.307, *P*<0.001; Fig. 2c), *P2ry6* (*F*_7,36_=7.586, *P*<0.001; Fig. 2f) and *P2ry12* (*F*_7,36_=9.544, *P*<0.001; Fig. 2g). We found no change in the mRNA levels for GCR encoded by *Nr3c1* gene (*F*_7,36_=0.347, *P*=0.926; Fig. 2b), *P2rx4* (*F*_7,36_=1.325, *P*=0.267; Fig. 2d) or *P2rx7* (*F*_7,36_=0.809, *P*=0.586; Fig. 2e) in the ipsilateral side of the spinal cord in male sham+PSL mice; moreover, their mRNA levels did not show significant time-dependent variations. A significant time-dependent variation in CORT levels was also detected in the spinal cord of male sham+PSL mice (Supplementary Fig. 2; Supplementary Method). Time-dependent increases and decreases in CORT levels in the spinal cord were similar to those observed in plasma. Although the time-dependent variations in CORT levels in the spinal cord was dampened in male ADX+PSL mice (Supplementary Fig. 2), adrenalectomy had a negligible effect on the PSL-induced increase in the number of Iba1-positive cells, as well as the expression of neuropathic pain-related genes in the ipsilateral side of the spinal cord. We also found an increase in the mRNA levels for *Irf8*, *P2ry6* and *P2ry12* in the inpsilateral spinal cord of female sham+PSL mice, and the PSL-induced molecular alterations were not modified by adrenalectomy (Supplementary Fig. 3). These results indicate that temporal elevations in glucocorticoid levels cause the diurnal oscillations in the threshold of pain hypersensitivity without affecting morphological or molecular alterations in spinal microglia.

### Glucocorticoids enhance ATP release from the spinal cord

**Figure 3:**
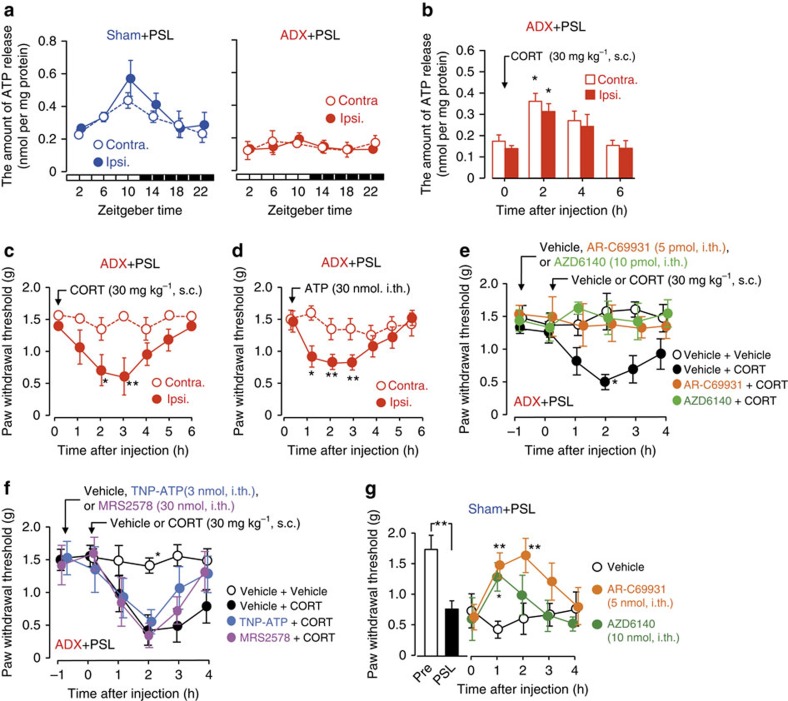
Glucocorticoids decrease the threshold of mechanical allodynia in PSL mice via P2Y_12_ receptor in the ipsilateral spinal cord. (**a**) Diurnal oscillations in ATP release from spinal slices were abrogated by adrenalectomy (means±s.e.m.; *n*=5–6). Significant time-dependent variations are observed in ATP release from the ipsilateral (Ipsi.) (*F*_5,30_=2.552, *P*=0.035; ANOVA) and contralateral (Contra.) sides (*F*_5,30_=2.049, *P*=0.048; ANOVA). (**b**) Subcutaneous (s.c.) administration of CORT (30 mg kg^−1^) to male ADX+PSL mice enhances ATP release from spinal slices (means±s.e.m.; *n*=5). *P<0.05 significantly different from the basal level (*F*_3,16_=3.755, *P*=0.042 for Ipsi.; *F*_3,16_=4.933, *P*=0.018 for Contra.; ANOVA with Tukey–Kramer's PHT). (**c**) Administration of CORT (30 mg kg^−1^, s.c.) to male ADX+PSL mice decreases the withdrawal threshold of the ipsilateral hindpaw (means±s.e.m.; *n*=6). **P<0.01; *P<0.05 significantly different from the basal level (*F*_6,35_=2.356, *P*=0.042; ANOVA with Tukey–Kramer's PHT). (**d**) Intrathecal (i.th.) injection of ATP (30 nmol per mouse) to male ADX+PSL mice decrease the withdrawal threshold of the ipsilateral hindpaw (means±s.e.m.; *n*=8). **P<0.01; *P<0.05 significantly different from the basal level (*F*_6,49_=3.964, *P*=0.002; ANOVA with Tukey–Kramer's PHT). (**e**) The P2Y_12_ receptor antagonists AR-C69931 (5 pmol per mouse, i.th.) or AZD6140 (10 pmol per mouse, i.th.) prevent CORT (30 mg kg^−1^, s.c.)-induced pain hypersensitivity in male ADX+PSL mice (means±s.e.m.; *n*=5–6). *P<0.05; significantly different from other groups (*F*_23,90_=1.012, *P*=0.046; ANOVA with Tukey–Kramer's PHT). (**f**) Effects of antagonist P2X_1–4_ receptors TNP-ATP (3 nmol per mouse, i.th.) or antagonist P2Y_6_ receptor MRS2578 (30 nmol per mouse, i.th.) on CORT (30 mg kg^−1^, s.c.)-induced pain hypersensitivity in male ADX+PSL mice (means±s.e.m.; *n*=5–6). *P<0.05; significantly different from other groups (*F*_23,93_=1.987, *P*=0.014; ANOVA with Tukey–Kramer's PHT). (**g**) The withdrawal threshold of the ipsilateral hindpaw of male sham+PSL mice after the injection of AR-C69931 (5 pmol per mouse, i.th.) or AZD6140 (10 pmol per mouse, i.th.) (means±s.e.m.; *n*=5–6), **P<0.01, *P<0.05 significantly different from vehicle (0.01% DMSO, 0.01% ethanol in PBS)-treated groups (*F*_14,75_=1.965, *P*=0.033; ANOVA with Tukey–Kramer's PHT). Time of drug administration was set at ZT10. All experiments were performed on day 7 after nerve injury.

Extracellular ATP in the spinal cord has been implicated in the development and maintenance of pain hypersensitivity after nerve injury^10^,^11^,^12^,^13^. Therefore, we also investigated whether temporary changes in glucocorticoid levels affected extracellular ATP release in the spinal cord of PSL mice. We prepared contralateral and ipsilateral spinal slices from L4-L5 lumbar segments of sham+PSL and ADX+PSL male mice at six different time points. The amount of ATP in the perfusate of the slices after incubation for 20 min was measured as an increase in ATP release. The increased amount of ATP was normalized to protein contents of the slices. The release of ATP from spinal slices prepared from male sham+PSL mice exhibited a significant diurnal oscillation with a peak from the late light phase to the early dark phase (*F*_5,30_=2.552, *P*=0.035 for the ipsilateral side; *F*_5,30_=2.049, *P*=0.048 for the contralateral side; Fig. 3a, left panel). Similar time-dependent variations were also detected in ATP concentrations in the cerebrospinal fluid (CSF) of sham+PSL mice (*P*<0.05; Supplementary Fig. 4a; Supplementary Method). This may have been due to diurnal variations in the release of ATP from cells in the spinal cord. The rhythmic pattern of spinal ATP release in male sham+PSL mice resembled the overall oscillation in plasma and spinal CORT levels (Fig. 1b upper panel and Supplementary Fig. 2). In contrast, the oscillation in ATP release from spinal slices of male ADX+PSL mice was severely dampened (Fig. 3a, right panel); the release of ATP from both the ipsilateral and contralateral sides remained low throughout the day (*F*_5,28_=1.158, *P*=0.369 for the ipsilateral side; *F*_5,28_=0.492, *P*=0.779 for the contralateral side). Time-dependent variations in ATP concentrations in the CSF were also diminished in female ADX+PSL mice (Supplementary Fig. 4b). Since the single subcutaneous administration of CORT (30 mg kg^−1^) to male ADX+PSL mice significantly enhanced ATP release from the ipsilateral and contralateral spinal slices (*F*_3,16_=3.755, *P*=0.042 for the ipsilateral side, *F*_3,16_=4.933, *P*=0.018 for the contralateral side; Fig. 3b), diurnal changes in glucocorticoid levels appear to govern the time-dependent oscillations in extracellular ATP release in the spinal cord.

The administration of CORT to male ADX+PSL mice significantly enhanced ATP release from both the ipsilateral and contralateral sides of the spinal cord. However, the withdrawal threshold of the ipsilateral hindpaw only decreased significantly after the administration of CORT (*F*_6,35_=2.356, *P*=0.042 for the ipsilateral side; *F*_6,35_=1.075, *P*=0.396 for the contralateral side; Fig. 3c). Due to the ability of glucocorticoids to enhance ATP release as well as produce mechanical allodynia in ADX+PSL mice, we also examined whether ATP was involved in the glucocorticoid-induced decrease in the threshold of pain hypersensitivity. The single intrathecal injection of ATP (30 nmol per mouse) to male ADX+PSL mice transiently, but significantly decreased the withdrawal threshold of the ipsilateral hindpaw (*F*_6,49_=3.964, *P*=0.002; Fig. 3d); however, ATP-induced decreases in the withdrawal threshold were not detected in the contralateral hindpaw (*F*_6,49_=0.518, *P*=0.792). Taken together, these results indicate the possibility that glucocorticoids decrease the threshold of mechanical allodynia in PSL mice by enhancing the extracellular release of ATP in the spinal cord.

To explore this possibility, we investigated whether CORT-evoked mechanical allodynia in ADX+PSL mice was attenuated by antagonists of purinergic receptors. In this experiment, all antagonists were intrathecally injected into ADX+PSL mice at 1 h before the administration of CORT (30 mg kg^−1^, s.c.). The withdrawal threshold of ispilateral hindpaw of male ADX+PSL mice after the CORT treatment was significantly decreased (*F*_23,90_=1.012, *P*=0.046; Fig. 3e), which was consistent with the results described above. The CORT-induced decrease in the pain threshold of the ipsilateral hindpaw in male ADX+PSL mice was prevented by pretreatment with the P2Y_12_ receptor antagonists, AR-C69931 (ref. 13) or AZD6140 (ref. 27) (*P*<0.05, respectively). On the other hand, neither 2′,3′-O-(2,4,6-trinitrophenyl) adenosine 5-triphosphate (TNP-ATP) (ref. 12), an antagonist of P2X1–4 receptors, nor MRS2578 (ref. 28), an antagonist of P2Y_6_ receptor, suppressed CORT-evoked mechanical allodynia in male ADX+PSL mice (Fig. 3f). The single intrathecal injection of AR-C69931 or AZD6140 also prevented PSL-induced decreases in the withdrawal threshold of ipsilateral hindpaw of male mice (*F*_14,75_=1.965, *P*=0.033; Fig. 3g). We also found that the P2Y_12_ receptor antagonists, AR-C69931, alleviated CORT- or PSL-induced mechanical allodynia in female mice (Supplementary Fig. 5). Since, the mRNA levels of *P2ry12* were significantly elevated in the ipsilateral spinal cord of both male and female ADX+PSL mice (Fig. 2g), P2Y12 receptors appeared to be involved in the glucocorticoid-induced decrease in the threshold of mechanical allodynia in ADX+PSL mice.

### Glucocorticoids induce exacerbation of mechanical allodynia via SGK-1

Glucocorticoids modulate the physiology of the cell by activating or repressing the expression of its target genes^29^. Therefore, we hypothesized that glucocorticoids caused diurnal oscillations in spinal ATP release by inducing specific genes. To screen for glucocorticoid-regulated genes whose expression in the spinal cord exhibited diurnal oscillations, we performed oligonucleotide microarray analyses using RNA isolated from the spinal cords of male sham+PSL or ADX+PSL mice at ZT10 and ZT22, times at which plasma glucocorticoid levels in sham+PSL mice peaked and declined, respectively (Fig. 1b). Three criteria were applied to select diurnal cycle-dependent glucocorticoid-regulated genes in the spinal cord. (1) Sham+PSL at ZT10>Sham+PSL at ZT22; (2) ADX+PSL at ZT10≒ADX+PSL at ZT22; and (3) Sham+PSL>ADX+PSL. From this analysis, we identified 25 genes as candidates for diurnal time-dependent glucocorticoid-regulated genes expressed in the spinal cord (Table 1). The glucocorticoid-regulated gene *Sgk-1* was included as a diurnal time-dependent spinal gene. Since typical glucocorticoid response elements are located in the upstream promoter region of both mouse and human *Sgk-1* genes, we focused on this gene to examine whether SGK-1 was involved in the glucocorticoid-regulated diurnal release of ATP in the spinal cord as well as time-dependent oscillations in the threshold of mechanical allodynia in PSL mice.

**Figure 4:**
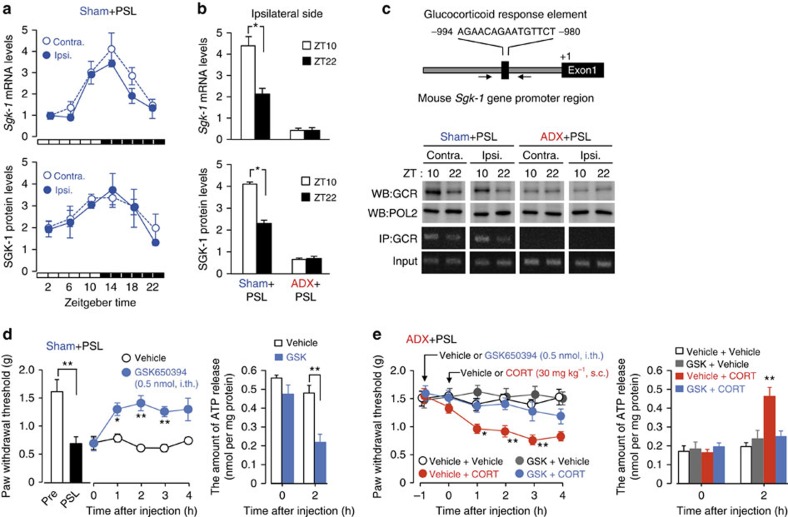
SGK-1 mediates the glucocorticoid-regulated diurnal oscillations in the threshold of mechanical allodynia in PSL mice. (**a**,**b**) Diurnal oscillations in the spinal expression of SGK-1 is dependent on glucocorticoid (means±s.e.m.; *n*=5). Significant time-dependent variations were observed in the levels of *Sgk-1* mRNA and its protein in the contralateral (Contra.) (*F*_5,24_=33.233, *P*<0.001 for mRNA; *F*_5,24_=4.808, *P*=0.006 for protein; ANOVA) and the ipsilateral (Ipsi.) sides (*F*_5,24_=14.996, *P*<0.001 for mRNA; *F*_5,24_=4.591, *P*=0.007 for protein; ANOVA). *P<0.05 significant difference between two time points (*F*_3,16_=105.464, *P*<0.001 for mRNA; *F*_3,16_=670.562, *P*<0.001 for protein; ANOVA with Tukey–Kramer's PHT). (**c**) The temporal binding profiles of the GCR to the *Sgk-1* promoter in the spinal cords of sham+PSL and ADX+PSL male mice. GCR and RNA polymerase 2 (POL2) in the nuclear fraction of spinal cords were detected by western blotting (WB). Immunoprecipitates (IP) with anti-GCR antibodies were subjected to a PCR analysis. Solid line arrows in the upper panel represent PCR amplification areas. Data are representative of three independent experiments. Full-size images of WB are presented in Supplementary Fig. 13. (**d**) The intrathecal (i.th.) injection of the SGK-1 inhibitor GSK650394 (0.5 nmol per mouse) alleviates pain hypersensitivity of the ipsilateral hindpaw in male sham+PSL mice and suppresses of spinal ATP release (means±s.e.m.; *n*=5), **P<0.01, *P<0.05 significantly different from vehicle-treated groups at the corresponding time points (*F*_9,40_=8.876, *P*<0.001 for pain threshold, *F*_3,16_=12.454, *P*<0.001 for ATP release; ANOVA with Tukey–Kramer's PHT). (**e**) The SGK-1 inhibitor GSK650394 (0.5 pmol per mouse, i.th.) prevents CORT (30 mg kg^−1^, s.c.)-induced pain hypersensitivity of the ipsilateral hindpaw in male ADX+PSL mice and suppresses spinal ATP release (means±s.e.m.; *n*=5) **P<0.01, *P<0.05 significantly different from the other groups (*F*_23,96_=3.599, *P*<0.001 for pain threshold, *F*_7,36_=5.735, *P*<0.001 for ATP release; ANOVA with Tukey–Kramer's PHT). The administration time of CORT or GSK650394 was set at ZT10. All experiments were performed on day 7 after nerve injury.

The expression of *Sgk-1* mRNA and its protein exhibited a significant diurnal oscillations in both the ipsilateral (*F*_5,24_=33.233, *P*<0.001 for mRNA; *F*_5,24_=4.808, *P*=0.006 for protein) and contralateral (*F*_5,24_=14.996, *P*<0.001 for mRNA; *F*_5,24_=4.591, *P*=0.007 for protein) sides of the spinal cord of male sham+PSL mice (Fig. 4a). However, oscillations were not detected in the ipsilateral (*P*=0.995 for mRNA, *P*=0.979 for protein) side of male ADX+PSL mice (Fig. 4b). Disruption of diurnal variation in *Sgk-1* mRNA levels was also detected in female ADX+PSL mice (Supplementary Fig. 6). The results of the chromatin immunoprecipitation analysis indicated that diurnal oscillations in the spinal expression of *Sgk-1* were associated with enhanced binding of the GCR to the promoter region of the gene (Fig. 4c). The induction of the spinal expression of *Sgk-1* and enhancements in GCR binding to the promoter region were also detected when male ADX+PSL mice were subcutaneously administered with 30 mg kg^−1^ CORT (Supplementary Fig. 7). The single intrathecal injection of the SGK-1 inhibitor GSK650394 (0.5 nmol per mouse) to male sham+PSL mice increased the threshold of mechanical allodynia (*F*_9,40_=8.876, *P*<0.001; Fig. 4d), and this was accompanied by the suppression of extracellular ATP release in the spinal cord (*F*_3,16_=12.454, *P*<0.001). Furthermore, the intrathecal injection of GSK650394 (0.5 nmol per mouse) to male ADX+PSL mice prevented the CORT-induced decrease in the threshold of mechanical allodynia (*F*_23,96_=3.599, *P*<0.001), as well as spinal ATP release (*F*_7,36_=5.735, *P*<0.001; Fig. 4e). Similar inhibitory effects of GSK650394 on CORT- or PSL-induced mechanical allodynia were also observed in female mice (Supplementary Fig. 5). These results suggest that the glucocorticoid-induced exacerbation of neuropathic pain hypersensitivity depends on SGK-1.

### SGK-1 mediates the glucocorticoid-enhanced ATP release from astrocytes

**Figure 5:**
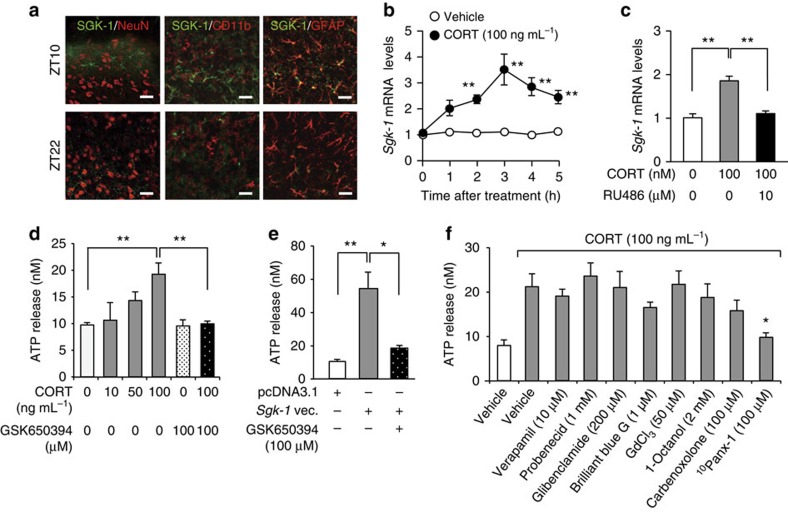
The expression of SGK-1 enhances ATP release from astrocytes. (**a**) Double immunofluorescence labelling of SGK-1 with NeuN, a marker of neurons; CD11b, a marker of microglia; and GFAP, a marker of astrocytes in the ipsilateral spinal slices of male sham+PSL mice at ZT10 and ZT22. SGK-1-positive cells are double-labelled (yellow) with GFAP at ZT10. This experiment was performed on day 7 after nerve injury. Scale bar, 10 μm. Data are representative of five independent experiments. (**b**) Time course of the CORT-induced expression of *Sgk-1* mRNA in primary cultures of astrocytes (means±s.e.m.; *n*=5). **P<0.01 significantly different from the basal level (*F*_11,48_=25.460, *P*<0.001, ANOVA with Tukey–Kramer's PHT). (**c**) The induction of *Sgk-1* mRNA by CORT (100 ng ml^−1^) is inhibited by 10 μM RU486 (means±s.e.m.; *n*=5). **P<0.01 significant difference between two groups (*F*_2,12_=55.500, *P*<0.001, ANOVA with Tukey–Kramer's PHT). (**d**) The SGK-1 inhibitor GSK650394 prevents the CORT-induced release of ATP from primary cultured astrocytes (means±s.e.m.; *n*=5). Cells were treated with CORT for 4 h in the presence or absence of 100 μM GSK650394. **P<0.01 significant difference between two groups (*F*_5,24_=6.175, *P*=0.017, ANOVA with Tukey–Kramer's PHT). (**e**) GSK650394 prevents the SGK-1-induced release of ATP from primary cultured astrocytes (means±s.e.m.; *n*=5). *Sgk-1*-expressing vector-transfected astrocytes were incubated in the presence or absence of 100 μM GSK650394. **P<0.01, *P<0.05 significant difference between two groups (*F*_2,12_=13.508, *P*=0.002, ANOVA with Tukey–Kramer's PHT). (**f**) Effects of inhibitors on candidate pathways for the CORT-induced release of ATP from astrocytes (means±s.e.m.; *n*=5–6 per group). Cells were treated with CORT for 4 h in the presence or absence of each inhibitor. *P<0.05 significant difference from CORT alone-treated cells (*F*_9,45_=3.094, *P*=0.005, ANOVA with Tukey–Kramer's PHT).

In an attempt to identify the types of cells expressing SGK-1, we performed double immunofluorescence labelling for SGK-1 and cell type-specific markers: for neurons, neuronal nuclei (NeuN); for microglia, CD11b; and for astrocytes, glial fibrillary acidic protein (GFAP). In the dorsal horn of the spinal cord in male sham+PSL mice, cells showing SGK-1 immunofluorescence were not double-labelled for NeuN or CD11b (Fig. 5a). Instead, most SGK-1-positive cells were double-labelled with GFAP, and their number in the dorsal horn was significantly higher at ZT10 than at ZT22 (51.78±6.11% for ZT10; 20.42±4.22% for ZT22; *n*=5, mean±s.e.m.; *t*_8_=4.031, *P*=0.004, unpaired *t*-test, two-sided). Consistent with previous observation^14^, the immunoreactivity of GFAP were significantly increased in the ipsilateral spinal cord of male sham+PSL mice, whereas the number of GFAP-positive cells were similar between the two time points (Supplementary Fig. 8). Therefore, SGK-1 in the spinal cord of PSL mice appears to be mainly expressed in astrocytes and exhibits diurnal variation. The treatment of primary cultures of astrocytes with 100 ng ml^−1^ CORT significantly induced the expression of *Sgk-1* mRNA (*F*_11,48_=25.461, *P*<0.001; Fig. 5b), and this was suppressed by a concomitant treatment with the GCR antagonist RU486 (*F*_2,12_=55.500, *P*<0.001; Fig. 5c). The treatment with CORT for 4 h also enhanced ATP release from astrocytes (*F*_5,24_=6.175, *P*=0.017; Fig. 5d). The enhancing effects of CORT appeared to be mediated by SGK-1, because the treatment of cells with GSK650394 resulted in the suppression of the CORT-induced release of ATP (*P*<0.01). The ability of SGK-1 to enhance ATP release from astrocytes was further confirmed by the transfection of cells with *Sgk-1*-expressing vectors (Fig. 5e). The amount of ATP released from *Sgk-1*-expressing vector-transfected cells was approximately five-fold higher than that from empty (pcDNA3.1) vector-transfected cells (*F*_2,12_=13.508, *P*=0.002). The enhancing effect of SGK-1 was also prevented in cells treated with GSK650394 (*P*<0.05).

### SGK-1 enhances ATP release from astrocytes via pannexin-1

Several possible pathways have been suggested for ATP release in astrocytes^30^,^31^. To identify which pathway is involved in the SGK-1-induced enhancement in ATP release, we tested the sensitivity of CORT-treated astrocytes to inhibitors on the proteins contributing to possible ATP-releasing pathways. As demonstrated above (Fig. 5d), a significant increase in ATP release from astrocytes was detected when cells were treated with 100 ng ml^−1^ CORT for 4 h (*P*<0.05, *F*_9,45_=3.094, *P*=0.005; ANOVA with Tukey–Kramer's PHT; Fig. 5f). We then investigated the effects of different inhibitors by adding them to media at concentrations that were previously reported to be effective. The CORT-induced enhancement in astrocytic ATP release was unaffected by the treatments with 10 μM verapamil (*P*=0.999) or 1 mM probenecid (*P*=0.998), which are known to inhibit the transporting activity of p-glycoprotein^32^ and multidrug resistance protein 1 (ref. 33), respectively. Similarly, the treatment of cells with 200 μM glibenclamide, an inhibitor of the cystic fibrosis transmembrane conductance regulator anion channel^34^, also failed to prevent the CORT-induced enhancement in ATP release (*P*=0.999). An inhibitor of the P2X_7_ receptor (ref. 35), brilliant blue G (1 μM), did not have a significant effect on the release of ATP (*P*=0.778). Max-ion channels are shown to be responsible for the hypotonicity-induced ATP release from mouse astrocytes^36^. However, the treatment of cells with 50 μM GdCl_3_, an inhibitor of max-ion channels, did not suppress the CORT-induced enhancement in ATP release (*P*=0.989). Although no significant effects were observed when cells were treated with 2 mM 1-octanol (*P*=0.971), an inhibitor of connexins^37^ or 100 μM carbenoxolone (*P*=0.786), an inhibitor of both connexins and pannexins^38^, the selective pannexin-1 inhibitor, ^10^Panx-1 (100 μM) (ref. 39), significantly inhibited CORT-enhanced astrocytic ATP release (*P*<0.05). Since ^10^Panx-1 also inhibited the enhancement effects of SGK-1 on the astrocytic ATP release (Supplementary Fig. 9a; Supplementary Method), we further investigated the ability of this channel to mediate ATP release in response to glucocorticoids.

**Figure 6:**
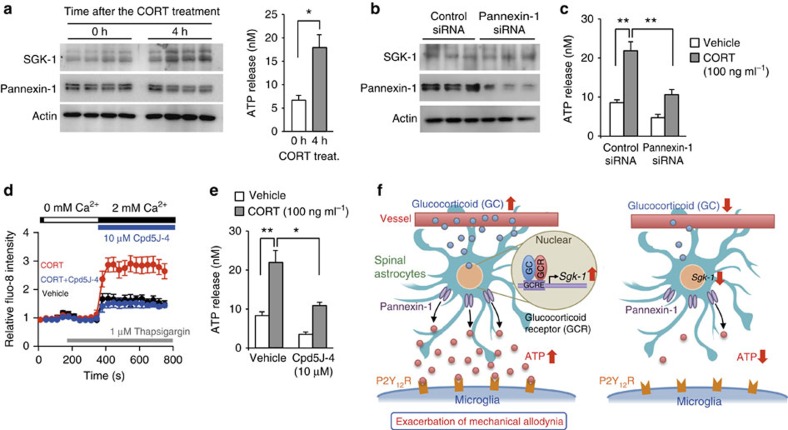
Induction of SGK-1 by glucocorticoid enhances ATP release from astrocytes via pannexin-1 hemichannels. (**a**) Negligible effects of CORT (100 ng ml^−1^) on the expression of pannexin-1 in the membrane fraction of astrocytes (left) and release of ATP (right). Cells are treated with 100 ng ml^−1^ for 4 h. CORT *P<0.05 significant difference between two groups (*n*=5, means±s.e.m.; *t*_8_=−5.445, unpaired *t*-test, two-sided). Full-size images are presented in Supplementary Fig. 14. (**b**) The downregulation of pannexin-1 in the membrane fraction of astrocytes. Cells are transfected with 150 pmol of siRNA against pannexin-1 or scrambled (Control) siRNA. Protein levels were assessed at 72 h after transfection. Full-size images are presented in Supplementary Fig. 15. (**c**) Effects of the downregulation of pannexin-1 on the CORT-induced release of ATP from astrocytes (means±s.e.m.; *n*=5 per group). Astrocytic ATP release was assessed 4 h after the CORT treatment. **P<0.01 significant difference between two groups (*F*_3,16_=19.130, *P*<0.001, ANOVA with Tukey–Kramer's PHT). (**d**) Tracing of the fluo-8 fluorescence intensity during and after Ca^2+^ depletion in astrocytes treated with 100 ng ml^−1^ CORT in the presence or absence of 10 μM Cpd5J-4 an Orai1 inhibitor (means±s.e.m.; *n*=8 per groups). Average fluorescence before the measurement is set to 1. (**e**) Effects of Cpd5J-4 on the CORT-induced release of ATP from astrocytes (means±s.e.m.; *n*=5 per group). Cells were treated with 100 ng ml^−1^ CORT in the presence or absence of Cpd5J-4 (10 μM) for 4 h. **P<0.01; *P<0.05 significant difference between two groups (*F*_3,16_=19.323, *P*<0.001, ANOVA with Tukey–Kramer's PHT). (**f**) Schematic diagram indicating the glucocorticoid-regulated diurnal oscillations in the threshold of mechanical allodynia in PSL mice. Temporal elevations in glucocorticoid levels induce the expression of SGK-1 in spinal astrocytes, thereby enhancing extracellular ATP release through pannexin-1 hemichannels. ATP released from astrocytes binds to P2Y_12_ receptors on activated microglia. ATP-stimulated P2Y_12_ receptors induce downstream events, which result in a decrease in the threshold of mechanical allodynia.

The treatment of primary cultured astrocytes with 100 ng ml^−1^ CORT for 4 h did not change the protein levels of pannexin-1 (Fig. 6a) despite enhancing ATP release (*t*_8_=3.087, *P*=0.021; unpaired *t*-test, two-sided). On the other hand, the downregulation of this hemichannel by siRNA (Fig. 6b) significantly attenuated the CORT-induced enhancement in ATP release from astrocytes (*F*_3,16_=19.130, *P*<0.001; Fig. 6c). The transfection of primary cultured astrocytes with *Sgk-1-*expressing vectors had a negligible effect on the protein levels of pannexin-1 (Supplementary Fig. 9b). Therefore, we considered that CORT-induced SGK-1 to enhance the release of ATP from astrocytes by opening of pannexin-1 hemichannels.

The opening of pannexin-1 is gated by cellular signals such as membrane depolarization and intracellular calcium^40^. Since SGK-1 has the ability to increase the activity of Orai1 (ref. 41), the pore-forming unit of store-operated calcium entry, we investigated whether the inhibition of Orai1-mediated intracellular calcium entry also suppressed the CORT-enhanced release of ATP from astrocytes. The results of a Fluo-8 fluorescence analysis revealed that the treatment of astrocytes with 100 ng ml^−1^ CORT-enhanced store-operated calcium entry (Fig. 6d), and the enhancing effects of CORT were suppressed by treating of cells with 10 μM Cpd5J-4, an inhibitor on Orai1 (ref. 42). This Orai1 inhibitor also prevented the release of ATP from CORT-treated cells (*F*_3,16_=19.323, *P*<0.001; Fig. 6e). Cpd5J-4 exerted similar inhibitory effects on intracellular calcium entry and ATP release were also observed in SGK-1 overexpressing astrocytes (Supplementary Fig. 10; Supplementary Method). These results suggest that glucocorticoid-induced SGK-1 enhances ATP release from astrocytes through pannexin-1 hemichannels gated by store-operated calcium entry.

## Discussion

Although the extracellular ATP release in the spinal cord is considered to activate the microglia and produce neuropathic pain behaviour^9^, the results of the present study suggest that the glucocorticoid-induced release of ATP from spinal astrocytes is involved in the diurnal decrease in the threshold of mechanical allodynia in PSL mice. Astrocytes play an active role in cell-to-cell signalling in the central nervous system through their release of gliotransmitters, which form neuron–glia and glia–glia networks^31^,^43^. Astrocytes have been shown to release ATP in response to mechanical stress, osmotic swelling, the deprivation of extracellular calcium, and stimulations with noradrenaline, nitrogen monoxide and uridine triphosphate^31^. In addition to these endogenous compounds, we found that glucocorticoids enhanced astrocytic ATP release by inducing the expression of SGK-1. A previous study reported that SGK-1 was expressed in both neurons and astrocytes^44^. The L5 spinal nerve ligation (SNL) of male Sprague–Dawley rats is known to induce the phosphorylation of SGK-1 in the ipsilateral dorsal horn without changing its total protein level^45^. Although there is no direct evidence for SGK-1 expression in neural cells in the spinal cord, phosphorylated SGK-1 has been suggested to be associated with mechanical allodynia in SNL rats through the activation of glutamatergic *N*-methyl-D-aspartate receptors^45^. In the present study, we used a PSL model in mice, because they exhibited diurnal oscillations in the threshold of mechanical allodynia^46^. The results of immunofluorescence analysis indicated that SGK-1 in the spinal cord of PSL mice was expressed in astrocytes rather than in neurons. Glucocorticoids have been shown to induce the expression of SGK-1 more selectively in astrocytes than in neurons^44^. We detected coincident immunoreactivity for SGK-1 and GFAP in the spinal cord of sham+PSL mice around the peak time of diurnal glucocorticoid secretion. These results indicate that the diurnal expression of SGK-1 in the spinal cord is attributable to its temporal accumulation in astrocytes.

The times of the day-dependent changes in the adrenal secretion of glucocorticoids are controlled by the master circadian clock, which is located in the SCN of the hypothalamus, and, in turn, coordinates various biological processes in peripheral tissues in a diurnal cycle-dependent manner^19^,^20^,^21^. The most parsimonious explanation for our results is that temporal elevations in glucocorticoid levels induce the expression of SGK-1 in spinal astrocytes, thereby enhancing extracellular ATP release through pannexin-1 hemichannels (Fig. 6f). The glucocorticoid-controlled diurnal release of ATP was detected in both contralateral and ipsilateral spinal cords of sham+PSL mice, whereas the number of microglia and expression of the *P2ry12* gene increased only in the ipsilateral side. Consequently, the glucocorticoid-enhanced release of ATP from astrocytes may stimulate P2Y12 receptors on microglia in the ipsilateral spinal cord, which then evoke mechanical allodynia in the nerve-injured hindpaw during a certain time of day.

SGK-1 is a member of the AGC kinase family and participates in the regulation of cell physiology through the phosphorylation of its target proteins^47^. Since elevations in the abundance of the SGK-1 protein in cultured astrocytes had negligible effects on the membrane expression of pannexin-1, this kinase appears to enhance extracellular ATP release through the opening of pannexin-1 hemichannels. The opening of pannexin-1 is known to be gated by various cellular signals^40^. As described above, SGK-1 has the ability to increase the activity of Orai1 (ref. 41). In fact, an inhibitor of Orai1 significantly suppressed the SGK-1-enhanced release of ATP from astrocytes, and this inhibitor also prevented the store-operated calcium entry. Therefore, we suggest that astrocytic ATP release by this mechanism may be responsible for the glucocorticoid-induced decrease in the threshold of mechanical allodynia in PSL mice.

The involvement of GCR in the development of mechanical allodynia has been demonstrated in rats following chronic constriction nerve injury^48^. The development of pain hypersensitivity in chronic constriction nerve injury rats has been implicated in the increase in the spinal expression of GCR, which is induced by interleukin-6 and protein kinase C. We also detected significant elevations in GCR mRNA levels in the ipsilateral spinal cord of male PSL model rats (Supplementary Fig. 11; Supplementary Method); however, an increase in GCR mRNA levels was not observed in the spinal cords of PSL mice (Fig. 2b). It is therefore possible that differences among animal species is reflected in the distinct expression patterns of GCR in the spinal cords of nerve-injured rats and mice. This may also be applicable to the absence of significant alterations in the spinal expression of the P2X4 receptor in PSL mice, because we observed elevations in the mRNA levels of both *P2rx4* and *P2ry12* in the ipsilateral spinal cord in male PSL rats (Supplementary Fig. 11). Consistent with previous findings in rats following SNL^12^,^13^, the development of pain hypersensitivity in PSL rats also appears to be associated with the activation of P2X_4_ and P2Y_12_ receptors in the spinal cord. Alternatively, we detected elevations in *P2rx4* mRNA levels in the ipsilateral spinal cord of C57BL mice following L4 spinal nerve transection (Supplementary Fig. 12; Supplementary Method). The transection of spinal nerves also tended to increase *P2rx4* mRNA expression in the ipsilateral spinal cord of ICR mice. In addition to the animal species and strain, the spinal expression of the P2X_4_ receptor may also be dependent on the nerve injury procedures.

A recent study has demonstrated that microglial involvement in mechanical allodynia is dependent on sex^49^. Nerve-injured female mice achieve similar levels of pain hypersensitivity as those in males, but this may be caused by proalgesic mediators released from T cells^49^. We also found that diurnal variation in the threshold of pain hypersensitivity of female sham+PSL mice was dependent on diurnal oscillation in glucocorticoid levels (Supplementary Fig. 1). Glucocorticoids also play a key role in conveying circadian signals from the SCN to the immune system^50^. Diurnal variations in plasma glucocorticoid levels show a negative correlation with 24-h changes in blood lymphocyte counts^51^. Elevations of glucocorticoid levels decrease the number of lymphocytes in the blood by enhancing the migration of these cells to tissues^52^. Time-dependent elevations in plasma CORT levels may also enhance the infiltration of immune cells into the inflammatory sites during a certain time of day, which causes diurnal variations in pain hypersensitivity in nerve-injured female mice. In addition to this possible mechanism, ATP release in the spinal cord appears to be partially involved in the mechanism underlying PSL-induced mechanical allodynia in female mice. As observed in males, female sham+PSL mice also exhibited diurnal variations in ATP concentrations in the CSF, accompanied by elevations in *P2ry12* mRNA levels in the ipsilateral spinal cord (Supplementary Fig. 3 and Fig. 4). Although the anti-allodynic effects were less as compared with those observed in males, the P2Y12 receptor antagonist AR-C69931 or SGK-1 inhibitor GSK650394 alleviated CORT- and PSL-induced mechanical allodynia in female mice (Supplementary Fig. 5). Taken together, glucocorticoids may also govern diurnal variations in pain hypersensitivity in female PSL mice by controlling extracellular ATP release in the spinal cord and activation of the P2Y_12_ receptor.

In clinical situation, high doses of synthetic glucocorticoids are used in the management of neuropathic pain^53^. Synthetic glucocorticoids are considered to reduce the symptoms of neuropathic pain through their anti-inflammatory and immunosuppressive actions. On the other hand, the role of endogenous glucocorticoids in nerve injury-induced pain hypersensitivity had been enigmatic. Under certain conditions, physiological levels of endogenous glucocorticoids have enhancing effects on the immune system^54^, while similar levels of glucocorticoids suppress autoimmune and inflammatory reactions under other conditions. These differential effects of glucocorticoids on the immune system may be due to the differences in the duration of their exposure^55^.

Diurnal rhythms in pain hypersensitivity have been demonstrated clinically^1^,^2^,^3^,^4^,^5^,^6^. However, the temporal pattern of the exacerbation of pain is distinct in a manner that is dependent on the cause of the pain. For example, cancer-associated pain is exacerbated around the morning^1^ or during the daytime^2^, but the intensity of spontaneous pain in patients with diabetic neuropathy or post-herpetic neuralgia has been reported to decrease during the night^4^. Although the rhythmic phase of adrenal glucocorticoid secretion is controlled by the circadian clock machinery, the secretion pattern is sometimes modulated under pathological conditions^56^,^57^. The understanding of such patterns is necessary for research on the mechanisms and triggers of chronic pain, and for appropriate patient care, including optimal timing of analgesic treatment. Our findings reveal an unappreciated circadian machinery affecting pain hypersensitivity caused by peripheral nerve injury, and also provide novel approaches to the management of chronic pain.

## Methods

### Animals and treatments

Male ICR mice (5–6 weeks old) were housed in groups (from 6 to 10 per cage for mice and from 3 to 4 per cage for rats) in a light-controlled room (lights on from ZT0 to ZT12) at 24±1 °C and humidity of 60±10% with food and water *ad libitum*. We prepared ADX mice under sodium pentobarbital (40 mg kg^−1^, i.p.) and isoflurane anaesthesia. The adrenals were removed via a dorsal approach using an aseptic technique. ADX mice were given 0.9% NaCl to drink during the duration of the experiment. Sham adrenalectomy was performed using the same procedure to expose the adrenals without their removal. Mouse models of PSL were also prepared under anaesthesia. The right thigh was shaved and the sciatic nerve was exposed through an incision. Half of the nerve was tightly ligated with 8-0 silk thread and the wound was sutured (Ipsilateral side; right hindpaw). The sciatic nerve of the left hindpaw was also exposed by the same procedure; however, the wound was sutured without nerve ligation (Contralateral side; left hindpaw). ADX+PSL mice were anaesthetized and an osmotic pump (Model 2001, Alzet, Alza Corp., Palo Alto, CA) was implanted in the subcutaneous pocket over the back. These osmotic pumps were designed to deliver a flow rate of 1 μl h^−1^ for 7 days and were filled with CORT dissolved in water and propylene glycol (1:1 volume) with 5% DMSO and 5% ethanol. AR-C69931 (AdooQ Bioscience, Irvine, CA), AZD6140 (Cayman Chemical, Ann Arbor, MI), and TNP-ATP (Sigma-Aldrich, St Louis, MO) were dissolved in phosphate-buffered saline (PBS) with 0.01% DMSO and 0.01% ethanol. A solution of MRS2578 (Sigma-Aldrich) was prepared by dissolving in 5% DMSO and 95% olive oil. Drugs were intrathecally injected using a 30-gauge needle. A volume of 10 μl drug solution was injected over 30 s. All experimental procedures were performed under the approval and guidelines of Kyushu University.

### Measurement of plasma CORT levels

Blood samples were drawn by orbital sinus collection and plasma samples were obtained after centrifugation at 3,000 r.p.m. for 3 min. CORT concentrations were measured using a corticosterone enzyme immunoassay kit (Cayman Chemical) validated for mouse plasma samples according to the manufacturer's protocol.

### Assessment of mechanical allodynia

To assess mechanical allodynia, mice were placed individually in an opaque plastic cylinder, which was placed on a wire mesh and habituated for 0.5 h to allow acclimatization to the new environment. Calibrated von Frey filaments (0.02–2.0 g, North Coast Medical) were then applied to the plantar surfaces of the hindpaws of mice. The paw withdrawal threshold was evaluated using the up-down method^12^,^13^. Observers were blinded to the drug treatment.

### Quantitative RT-PCR analysis

To extract mRNA from the spinal cord of mice, contralateral and ipsilateral spinal slices from lumbar segments L4 to L5 were prepared. RNA was extracted using RNAiso reagent (Takara Bio Inc., Osaka, Japan). cDNA was synthesized using a ReverTra Ace qPCR RT Kit (Toyobo, Osaka, Japan) and amplified by PCR. A real-time PCR analysis was performed on diluted cDNA samples using the THUNDERBIRDTM SYBR qPCR Mix (Toyobo) with the 7500 Real-time PCR system (Applied Biosystems). Data were normalized using ‘18s' and ‘β-actin' mRNAs as a controls because spinal expression was constant throughout the day. Primer sequences were listed in Supplementary Table 1.

### Western blotting

Nuclear and cytosolic fractions of the spinal cords of mice were prepared at six time points. Membrane and cytosolic fractions of cultured astrocytes were also prepared by the same procedure. A total of 20 μg protein lysates was then resolved by 10% SDS-PAGE, transferred to a PVDF membrane, and probed with rabbit monoclonal antibodies against SGK-1 (1:1,000; ab32374, Abcam, Cambridge, UK), GCR (1:1,000; sc1002, Santa Cruz Biotechnology, Santa Cruz, CA), Pannexin-1 (1:1,000; ab124131, Abcam), Actin (1:1,000; sc1616-HRP, Santa Cruz Biotechnology) or POL2 (1:1,000; sc900, Santa Cruz Biotechnology). The specificities of antibodies for their target proteins have been validated in previous studies^58^,^59^,^60^. Specific antigen–antibody complexes were visualized using horseradish peroxidase-conjugated secondary antibodies and a chemiluminescence reagent (Nacalai Tesque, Kyoto, Japan).

### Measurement of ATP

To determine ATP concentrations in CSF, mice were anaesthetized using isoflurane and oxygen. CSF was collected by direct cisterna-magna puncture and diluted with 10-fold volume of artificial CSF (ACSF). The diluted samples were used for the ATP assay. Samples contaminated with erythrocytes or visibly haemolysed were not used for ATP measurements. To measure extracellular ATP release in the spinal cord, contralateral and ipsilateral spinal slices (500-μm-thick) from lumbar segments L4 to L5 were prepared and incubated in 300 μl of oxygenized ACSF for 20 min. Before and after incubation, 10 μl of ACSF was collected for the ATP assay. The residual slices in ACSF were homogenized and protein concentrations were measured using Lowry's method (DC protein assay; Bio-Rad). An increase in the amount of ATP released from the spinal slices was expressed as nmol mg^−1^ protein. To determine the release of ATP from cultured astrocytes, cells were seeded on 24-well culture plate and then incubated until they were in a semi-confluent state. For assessment of ATP release, culture media were replaced with 500 μl of assay butter containing 125 mM NaCl, 4.8 mM KCl, 5.6 mM D-glucose, 1.2 mM CaCl_2_, 1.2 mM KH_2_PO_4_, 1.2 mM MgSO_4_ and 25 mM HEPES (pH 7.4), and cells were incubated for 30 min. The assay buffer was also collected for the ATP assay. ATP levels were mesured using a bioluminescent ATP assay kit (Promega, Madison, WI). The ectonucleotidase inhibitor 6-*N*,*N*-diethyl-β-γ-dibromomethylene-D-adenosine-5-triphosphate FPL 67156 (ARL 67156 trisodium salt, Sigma-Aldrich) was added to ACSF or the assay buffer (100 μM final concentration) throughout the experiment to decrease ATP hydrolysis.

### Microarray analysis

Spinal slices (500-μm-thick) from lumber segments L4 to L5 were prepared from sham+PSL and ADX+PSL mice at ZT10 and ZT22. Total RNA (250 ng) was extracted from these slices using RNAiso (Takara Bio Inc.). The quality of the extracted RNA was analysed using an Agilent 2100 Bioanalyzer (Agilent Technologies, Santa Clara, CA). cRNA was amplified and labelled using a low-input quick amp labelling kit. Labelled cRNA was hybridized to a 44K Agilent 60-mer oligomicroarray (Whole Mouse Genome Microarray kit version 2.0) according to the manufacturer's instructions. All hybridized microarray slides were washed and scanned using an Agilent scanner. Relative hybridization intensities and background hybridization values were calculated using Agilent Feature Extraction software (version 9.5.1.1). Raw signal intensities and flags for each probe were calculated from hybridization intensities and spot information according to the procedures recommended by Agilent. The raw signal intensities of two samples were log2-transformed and normalized by a quantile algorithm in the ‘preprocessCore' library package of the Bioconductor software. This produced a gene expression matrix consisting of 55,681 probe sets, and differentially expressed genes between samples were selected by calculating *Z*-scores and ratios (non-log-scaled fold change) as follows: for upregulated genes: a *Z*-score of 2.0 or more and a ratio of twofold or more; and for downregulated genes: a *Z*-score of -2.0 or less and a ratio of 0.5 or less.

### Chromatin immunoprecipitation analysis

Cross-linked chromatin in the spinal cord was sonicated on ice, and nuclear fractions were obtained by centrifugation at 10 000*g* for 5 min. Supernatants were incubated with antibodies against GCR (1:500; sc1002, Santa Cruz Biotechnology) or rabbit IgG (1:500; sc66931, Santa Cruz Biotechnology). DNA was purified using a DNA purification kit (Promega) and amplified by PCR for the surrounding GRE in the 5′-flanking region of the *Sgk-1* gene. The primer sequences for amplification of the surrounding GRE were as follows: 5′-CTCACGTGTTCTTGGCATGG-3′ and 5′-TGGCCAAAACTAAGCAAGGC-3′. The quantitative reliability of PCR was evaluated by a kinetic analysis of the amplified products to ensure that signals were only derived from the exponential phase of amplification. This analysis also proceeded in the absence of an antibody or in the presence of rabbit IgG as negative controls. Ethidium bromide staining did not detect any PCR products in these samples.

### Immunofluorescent histochemical staining

Animals were anaesthetized and perfused transcardially with 4% paraformaldehyde in PBS, pH 7.4. The L4 spinal cord was removed and postfixed at 4 °C for 5 h. The postfixed spinal cord was transferred to 15% sucrose in PBS for 24 h and then 30% sucrose in PBS for 24 h. Floating transverse sections (30-μm-thick) were blocked in solution containing 10 % normal goat serum and 0.1% Triton X-100 for 2 h at 4 °C. The sections were then incubated for 48 h at 4 °C with primary antibodies against Iba1 (1:1,000; 019-19741, Wako Pure Chemicals), SGK-1 (1:1,000; ab32374, Abcam), NeuN (1:5,000, MAB377, Millipore Bioscience Research Reagents, Temecula, CA), CD11b (1:500; MCA711G, AbD Serotec, Oxford, UK) and GFAP (1:5,000; 13-0300, Invitrogen, Carlsbad, CA). The specificities of antibodies for their target proteins have been validated in previous studies^12^,^13^,^58^. After washing, the sections were incubated with a fluorescent-conjugated secondary antibody (Alexa 488 or Alexa 546, 1:1,000; Jackson ImmunoResearch, Laboratories, West Grove, PA) at 4 °C for 2 h. The sections were mounted with Vectashield (Vector Laboratories, Burlingame, CA). Fluorescent images were obtained with confocal fluorescence microscopy. The numbers of Iba1-positive microglial cells and GFAP-positive astrocytic cells were quantified with the Image J cell counter analysis tool in defined area of interest on the spinal cord dorsal horn. The score was blinded to sampling times and animal treatments.

### Preparation and treatment of primary cultures of astrocytes

A dissociated cell suspension was prepared from the cerebral cortex of neonatal mouse pups (2 days old). Cells were plated in tissue culture flasks at a concentration of 1 × 10^5^ cells per ml and incubated in DMEM supplemented with 10% fetal bovine serum (FBS) and antibiotics at 37 °C in a humidified 5% CO_2_ atmosphere for 72 h, which allowed the cells sufficient time to adhere and begin multiplying. Medium was changed every 72 h during the growth of cells. After incubating the primary cultures for 7–9 days, medium was changed completely and culture flasks were shaken to remove oligodendrocytes from the cultures. Fresh medium was added to the shaken flasks and the shaking process was repeated at least four times. The purification of primary cultures of astrocytes was confirmed by immunohistochemistry for the expression of GFAP. Astrocytes were treated with CORT for 4-h in the presence or absence of each inhibitor. The *Sgk-1*-expressing astrocytes were prepared by transfecting with Flag-tagged *Sgk-1*-expressing vectors, which were kindly provided by Dr Ono (Kyushu University). Vectors were transfected into cells using a squarewave electroporater (Model CUY21, Nepa Gene, Chiba, Japan). Transfection efficacy was confirmed by immunofluorescent staining by using anti-Flag monoclonal antibodies (F1804, Sigma-Aldrich). Approximately 70% of GFAP-positive cells merged with Flag-tagged SGK-1. Astrocytes were also transfected with 150 pmol siRNA against pannexin-1 (sc61288, Santa Cruz Biotechnology) using Lipofectamine 2000 reagent (Invitrogen Life Technologies) and used for experiments 72 h after transfection. The downregulated efficacy of this siRNA was confirmed, as shown in Fig. 6b.

### Calcium mobilization assay

Astrocytes (2 × 10^4^) were seeded on a 96-well plate. After 24 h, CORT was added to culture media at a final concentration of 100 ng ml^−1^. Four hours after the treatment, cells were loaded with 4 μM Fluo-8 AM (Abcam) in 100 μl of Hank's balanced salt solution containing 20 mM HEPES (pH 7.4) supplemented with 0.04% Pluronic F-127 and 1% FBS at 37 °C for 30 min, followed by a further incubation at room temperature for 30 min. Intracellular calcium mobilization was assessed by monitoring the fluorescence intensity (excitation at 490 nm and emission at 525 nm) using FlexStation 3 (Molecular Devices, Sunnyvale, CA, USA).

### Statistical and data analyses

The values presented are expressed as the means±s.e.m. The significance of the 24-h variations in each parameter was tested by ANOVA. The statistical significance of differences among groups was analysed by ANOVA followed by Tukey–Kramer's PHT. Equal variances were not formally tested. *P*<0.05 was considered significant. No statistical method was used to predetermine sample sizes; however, our sample sizes were similar to those reported in previous studies^13^,^36^,^46^. Experiments were not randomized.

### Data availability

All data supporting the results of the present study are included in the article, either in the main figures or the Supplementary Information Files. Microarray data were submitted to the Gene Expression Omnibus at the National Center for Biotechnology Information (accession # GSE65756).

## Additional information

**How to cite this article:** Koyanagi, S. *et al*. Glucocorticoid regulation of ATP release from spinal astrocytes underlies diurnal exacerbation of neuropathic mechanical allodynia. *Nat. Commun.* **7,** 13102 doi: 10.1038/ncomms13102 (2016).

## Supplementary Material

Supplementary Information Supplementary Figures 1-17, Supplementary Table 1, Supplementary Methods and Supplementary Reference.

## Figures and Tables

**Table 1:** Candidates for glucocorticoid-regulated genes in spinal cords of mice.

Genbank accession #	Gene symbol	Gene name	Sham+PSL ZT10/ZT22	ADX+PSL ZT10/ZT22	ADX+PSL/Sham+PSL	Location of GRE in upstream region
NM_010855	Myh4	Myosin, heavy polypeptide 4	213.1	Null	0.009	—
NM_001111293	Xlr5b	X-linked lymphocyte-regulated 5B	43.5	Null	0.063	—
NM_031493	Xlr5c	X-linked lymphocyte-regulated 5C	30.1	Null	0.043	—
NM_009394	Tnnc2	Troponin C2	17.3	1.3	0.133	—
NM_021285	Myl1	Myosin, light polypeptide 1	14.9	0.9	0.156	—
NM_001033350	Bank1	B-cell scaffold protein with ankyrin repeats 1	12.1	Null	0.089	—
NM_001045539	Xlr5a	X-linked lymphocyte-regulated 5A	11.6	Null	0.178	—
NM_175936	Vmn2r81	Vomeronasal 2, receptor 81	5.6	Null	0.126	—
NM_009148	Exoc4	Exocyst complex component 4	5.5	1.0	0.288	—
NM_010378	H2-Aa	Histocompatibility 2, class II antigen A, alpha	4.9	Null	0.163	—
NM_001033238	Cblb	Casitas B-lineage lymphoma b	4.5	1.5	0.474	—
NM_026428	Dcxr	Dicarbonyl L-xylulose reductase	4.4	0.9	0.349	—
NM_021365	Xlr4b	X-linked lymphocyte-regulated 4B	4.3	1.0	0.145	—
NM_007621	Cbr2	Carbonyl reductase 2	4.0	0.9	0.266	—
NM_009405	Tnni2	Troponin I, skeletal, fast 2	3.6	1.0	0.246	—
NM_001081106	Cytl1	Cytokine-like 1	3.2	1.0	0.327	—
NM_029568	Mfap4	Microfibrillar-associated protein 4	2.9	0.9	0.136	—
NM_007409	Adh1	Alcohol dehydrogenase 1	2.5	1.3	0.249	—
NM_001081643	Xlr3b	X-linked lymphocyte-regulated 3B	2.4	0.8	0.442	−
NM_001161845	Sgk-1	Serum- and glucocorticoid-inducible kinase-1	2.2	0.8	0.584	−994 to −980 bp
NM_015776	Mfap5	Microfibrillar-associated protein 5	2.0	0.8	0.084	—
NM_001099299	Ajap1	Adherens junction associated protein 1	1.8	0.9	0.603	—
NM_008788	Pcolce	Procollagen C-endopeptidase enhancer protein	1.8	1.0	0.250	—
NM_001012518	Ehmt1	Euchromatic histone methyltransferase 1	1.8	1.1	0.726	—
NM_178598	Tagln2	Transgelin 2	1.7	0.9	0.624	—

Values of ADX+PSL/Sham+PLS were calculated by mean expression levels in ADX+PSL mice between ZT10 and ZT22 being divided by the mean expression levels in sham+PSL mice between ZT10 and ZT22.
