# Supplementary material for: Glucocorticoid regulation of ATP release from spinal astrocytes underlies diurnal exacerbation of neuropathic mechanical allodynia
Source: Nat Commun. 2016 Oct 14;7:13102. doi: 10.1038/ncomms13102 (PMC5067584; doi:10.1038/ncomms13102)
Supplement: Supplementary Information — Supplementary Figures 1-17, Supplementary Table 1, Supplementary Methods and Supplementary Reference. [file ncomms13102-s1.pdf]

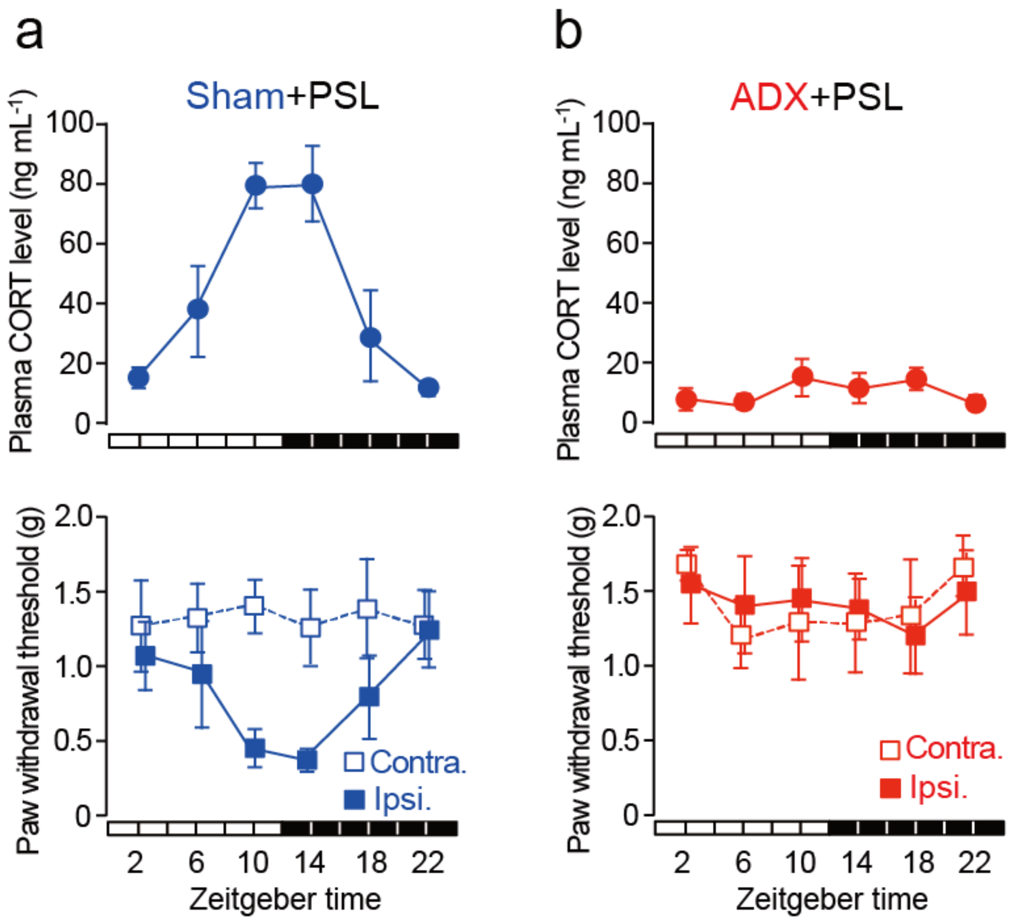

Supplementary Figure 1 GC regulates the diurnal oscillations in the threshold of mechanical allodynia in female PSL mice. Temporal profiles of plasma CORT levels and the paw withdrawal threshold in sham+PSL (a), ADX+PSL (b) female mice on day 7 after nerve injury (means ± s.e.m.; *n*=4). The withdrawal threshold of hindpaw was assessed by von Frey up-down method. Significant time-dependent variations are observed in plasma CORT levels in sham+PSL ($F_{5,20}$=5.764, *P*=0.002; ANOVA) mice. The withdrawal threshold of the ipsilateral hindpaw of sham+PSL mice also tends to show significant diurnal variation ($F_{5,20}$=2.330, *P*=0.079; ANOVA).

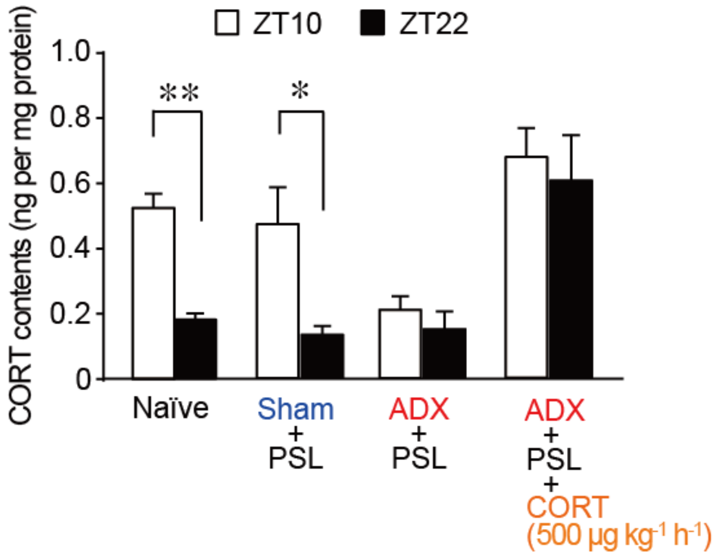

Supplementary Figure 2 Adrenalectomy abolishes diurnal variation in CORT contents in the spinal cord of male PSL mice. Temporal profiles of CORT levels in the spinal cord of naïve, sham+PSL, ADX+PSL, and CORT (500 μg kg^{-1} h^{-1})-administered male ADX+PSL mice on 7 days after nerve injury (means ± s.e.m.; *n*=4-5). The continuous administration of CORT (500 μg kg^{-1} h^{-1}) to ADX+PSL mice was conducted by the subcutaneous (s.c.) implantation of an osmotic minipump. **$P<0.01$, *$P<0.05$ significantly different in CORT content in the spinal cord between the two groups ($F_{7,30}$=11.047, $P<0.001$; ANOVA with Tukey-Kramer PHT).

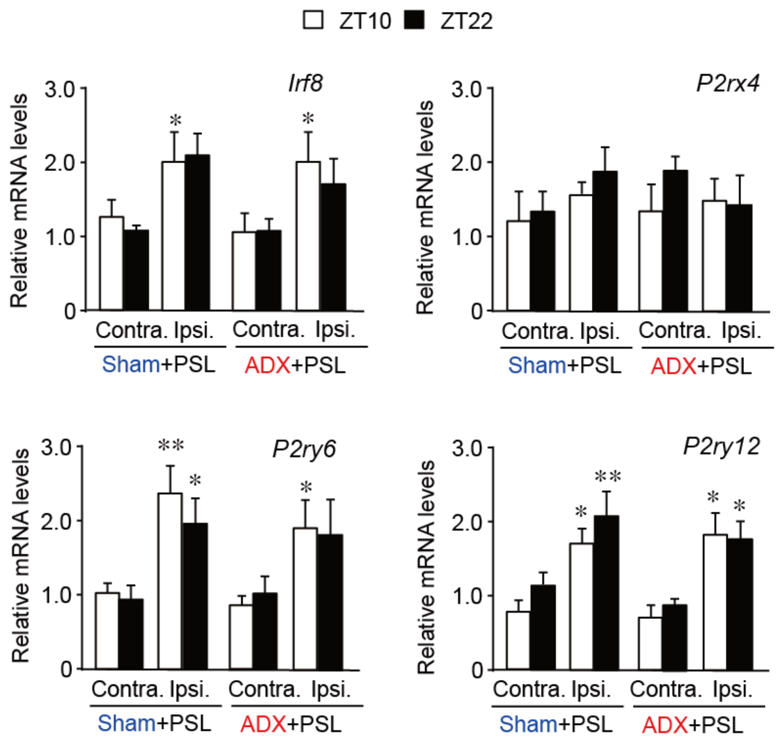

Supplementary Figure 3 Molecular alterations in the spinal cord of female PSL mice. The mRNA levels for Irf8, *P2rx4, P2rx6,* and *P2ry12* in the contralateral (Conta.) and ipsilateral (Ipsi.) spinal cords of sham+PSL and ADX+PSL female mice were assessed on day 7 after nerve injury (means ± s.e.m.; *n*=4-6). ***P*<0.01, **P*<0.05 significantly different from contralateral sides at the corresponding time ($F_{7,33}$=2.439, *P*=0.039 for *Irf8*; $F_{7,33}$=0.692, *P*=0.678 for *P2rx4*; $F_{7,33}$=3.344, *P*=0.008 for *P2ry6*; $F_{7,33}$=3.528, *P*=0.006 for *P2ry12*; ANOVA with Tukey-Kramer PHT).

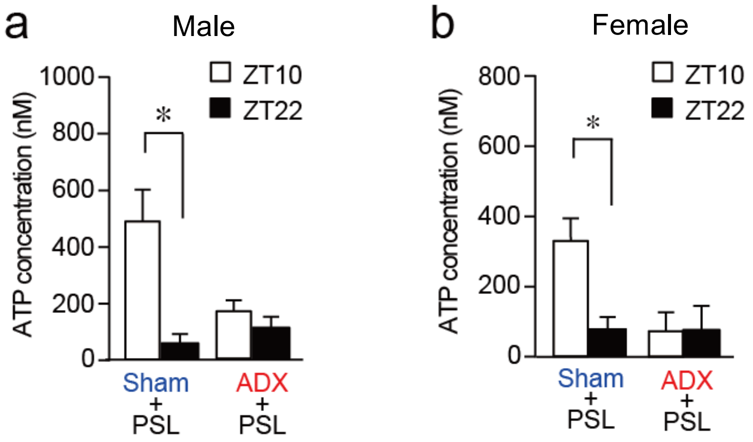

Supplementary Figure 4 Adrenalectomy abolishes diurnal variation in ATP concentration in the CSF of male and female PSL mice. (**a**), Temporal profiles of ATP concentration in the CSF of sham+PSL and ADX+PSL male mice on 7 days after nerve injury (means ± s.e.m.; *n*=5). **P*<0.05 significantly different in ATP concentration in the CSF between the two groups ($F_{3,16}$=3.483, *P*=0.010; ANOVA with Tukey-Kramer PHT). (**b**), Temporal profiles of ATP concentration in the CSF of sham+PSL and ADX+PSL female mice on day 7 after nerve injury (means ± s.e.m.; *n*=5 per group). **P*<0.05 significantly different between the two time points ($F_{3,16}$=5.357, *P*=0.009; ANOVA with Tukey-Kramer PHT).

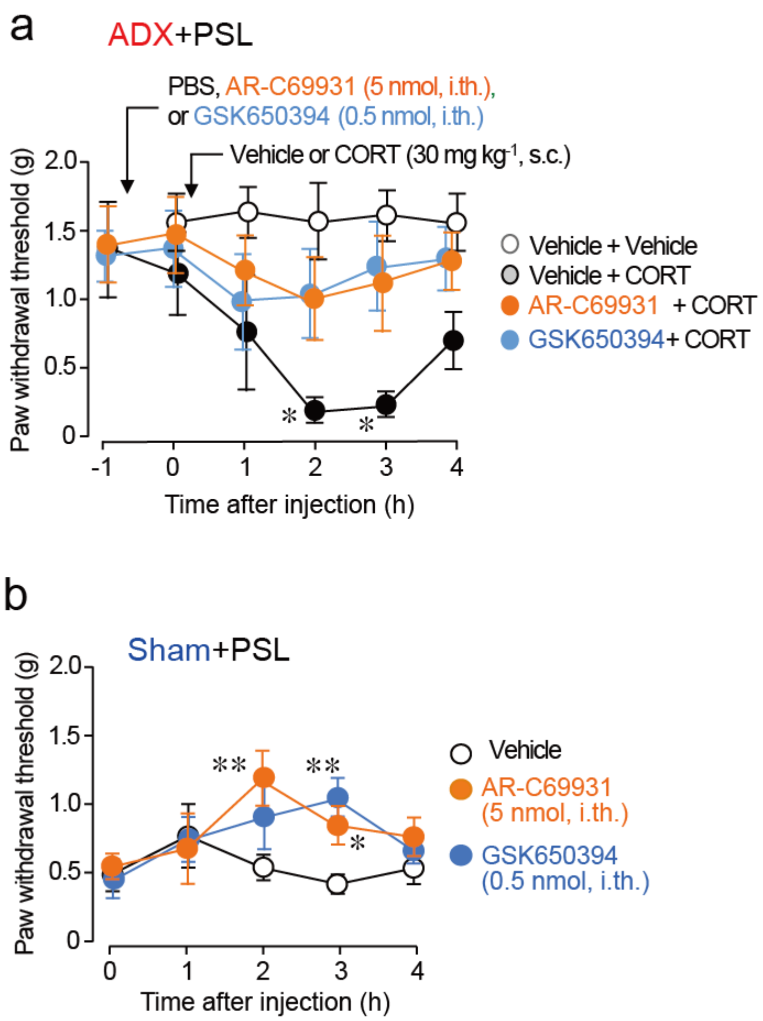

Supplementary Figure 5 The effects of SGK-1 inhibitor and P2Y$_{12}$ receptor antagonist on GC- and PSL-induced pain hypersensitivity in female mice. (**a**), The effects of SGK-1 inhibitor GSK650394 (0.5 nmol per mouse, i.th.) and P2Y$_{12}$ receptor antagonists AR-C69931 (5 pmol per mouse, i.th.) on the CORT (30 mg kg^{-1}, s.c.)-induced decrease in the withdrawal threshold of ipsilateral hindpaw of female ADX+PSL mice (means ± s.e.m.; *n*=5-6). **P*<0.05; significantly different from other groups at corresponding time points ($F_{22,109}$=1.678, *P*=0.044; ANOVA with Tukey-Kramer PHT). The administration time of CORT was set at ZT10. The drugs were injected at 1-h before CORT administration. (**b**), The withdrawal threshold of the ipsilateral hindpaw of sham+PSL female mice after intrathecal injection of GSK650394 (0.5 nmol per mouse, i.th.) or AR-C69931 (5 pmol per mouse, i.th.) (means ± s.e.m.; *n*=5-6). ***P*<0.01, **P*<0.05 significantly different from vehicle (0.01% DMSO, 0.01% ethanol in PBS)-treated groups at the corresponding time points ($F_{14,65}$=2.397, *P*=0.009; ANOVA with Tukey-Kramer PHT). All experiments were performed around ZT10-14 on day 7 after nerve injury.

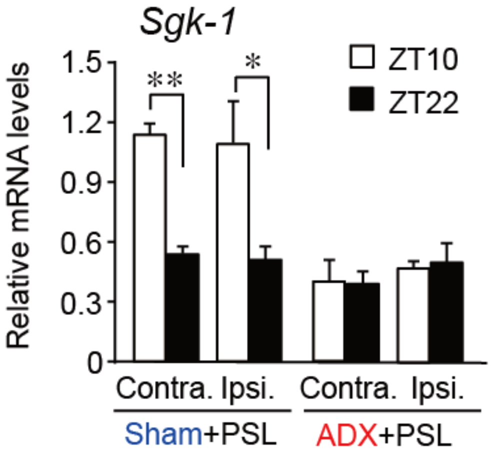

Supplementary Figure 6 Adrenalectomy abolishes diurnal variation in Sgk-1 mRNA expression in the spinal cord of female PSL mice. The mRNA levels of *Sgk-1* in the contralateral (Contra.) and ipsilateral (Ipsi.) spinal cords of sham+PSL and ADX+PSL female mice were assessed on day 7 after nerve injury (means ± s.e.m.; n=4-5). *P<0.01, *P<0.05 significantly different between the two groups ($F_{7,33}$=7.374, P<0.001; ANOVA with Tukey-Kramer PHT).

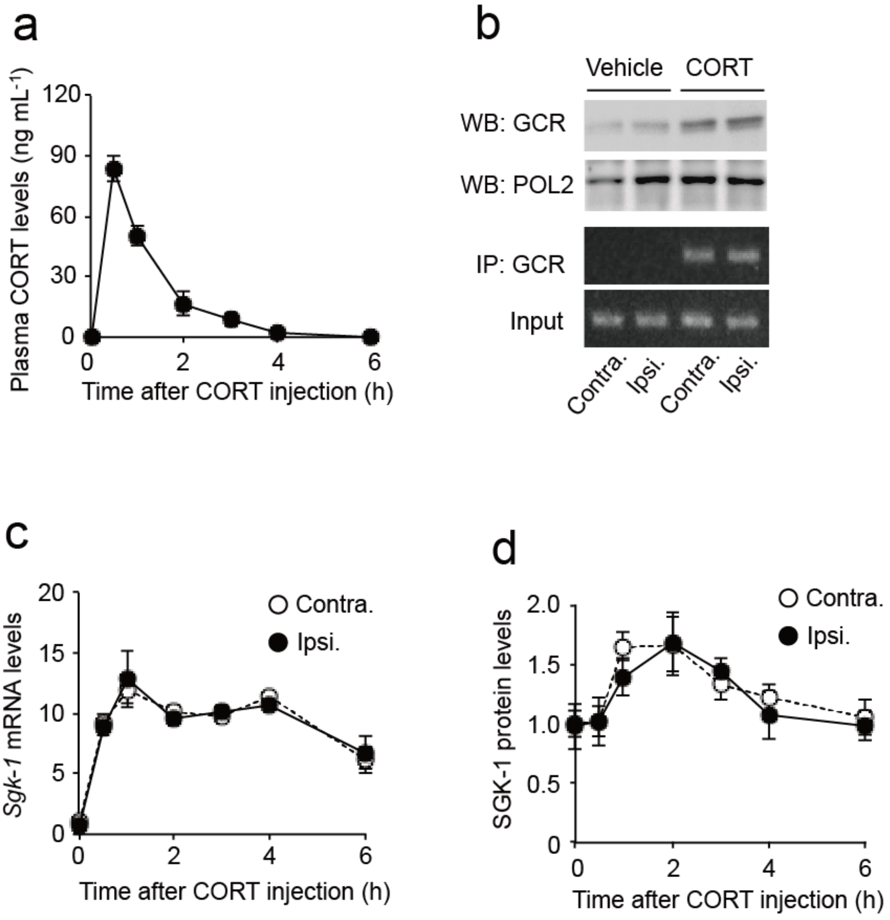

Supplementary Figure 7 GC-dependent expression of SGK-1 in spinal cord of male ADX+PSL mice. (**a**), Time course of plasma CORT levels in male ADX+PSL mice after a single subcutaneous (s.c.) administration of 30 mg kg^{-1} CORT (means ± s.e.m.; *n*=4) (**b**), Chromatin immunoprecipitation analysis for CORT (30 mg kg^{-1}, s.c.)-induced enhancement of glucocorticoid receptor (GCR) binding to the promoter region of *Sgk-1* gene in spinal cord of male ADX+PSL mice. The GCR binding was assessed 2 h after subcutaneous (s.c.) administration of 30 mg kg^{-1} CORT. GCR and RNA polymerase 2 (POL2) in the nuclear fraction of spinal cords were detected by western blotting (WB). Immunoprecipitates (IP) with anti-GCR antibodies were subjected to a PCR analysis as described in Fig. 4c. Data are representative of three independent experiments. Full size images are presented in Supplementary Figure 16. (**c, d**), Time course of the expression of *Sgk-1* mRNA (c) and its protein (d) in the ipsilateral (ipsi.) and contralateral (contra.) spinal cords of male ADX+PSL mice after subcutaneous administration of 30 mg kg^{-1} CORT (means ± s.e.m.; *n*=4). One-way ANOVA followed by Tukey-Kramer PHT reveals that both mRNA and protein levels of SGK-1 in the spinal cords are increased by CORT injection ($F_{13,42}$=13.019, P<0.001 for Sgk-1 mRNA and $F_{13,42}$=5.245, P<0.001 for SGK-1 protein). For all panels, the experiments were performed on day 7 after nerve injury.

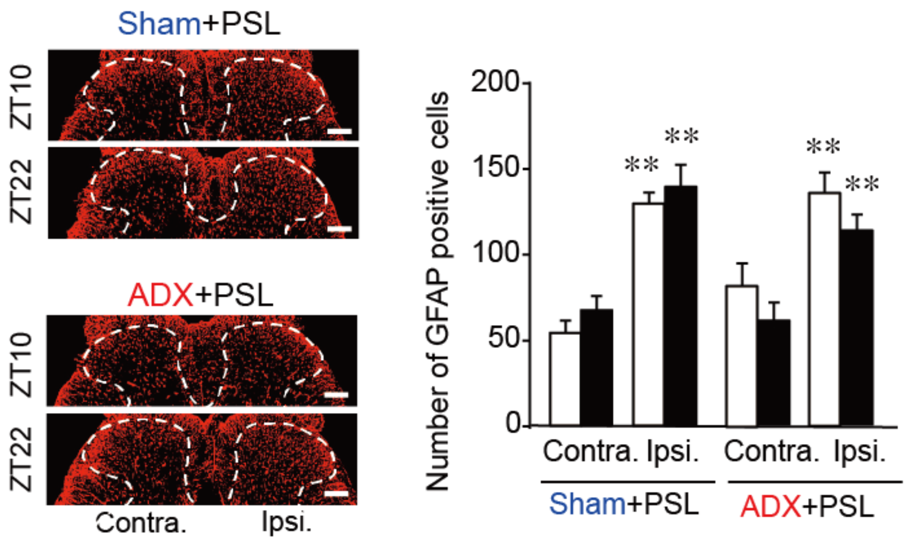

Supplementary Figure 8 Temporal profiles of GFAP-positive cells in the spinal cords of sham+PSL and ADX+PSL male mice. The right panel shows quantification of the number of GFAP-positive cells in the spinal cords of sham+PSL and ADX+PSL male mice on day 7 after nerve injury (means ± s.e.m.; *n*=6). The dorsal horn areas are surrounded with dashed line. Scale bar, 100 μm. Tukey-Kramer PHT shows that the number of GFAP-positive cells in the dorsal horn area of ipsilateral (Ipsi.) side of sham+PSL and ADX+PSL mice are significantly increased as compared with that in the contralateral (Contra.) side (**$P<0.01$), whereas no significant different is noted in the number of cells between the two time points ($F_{7,40}=17.172$, $P<0.001$; ANOVA).

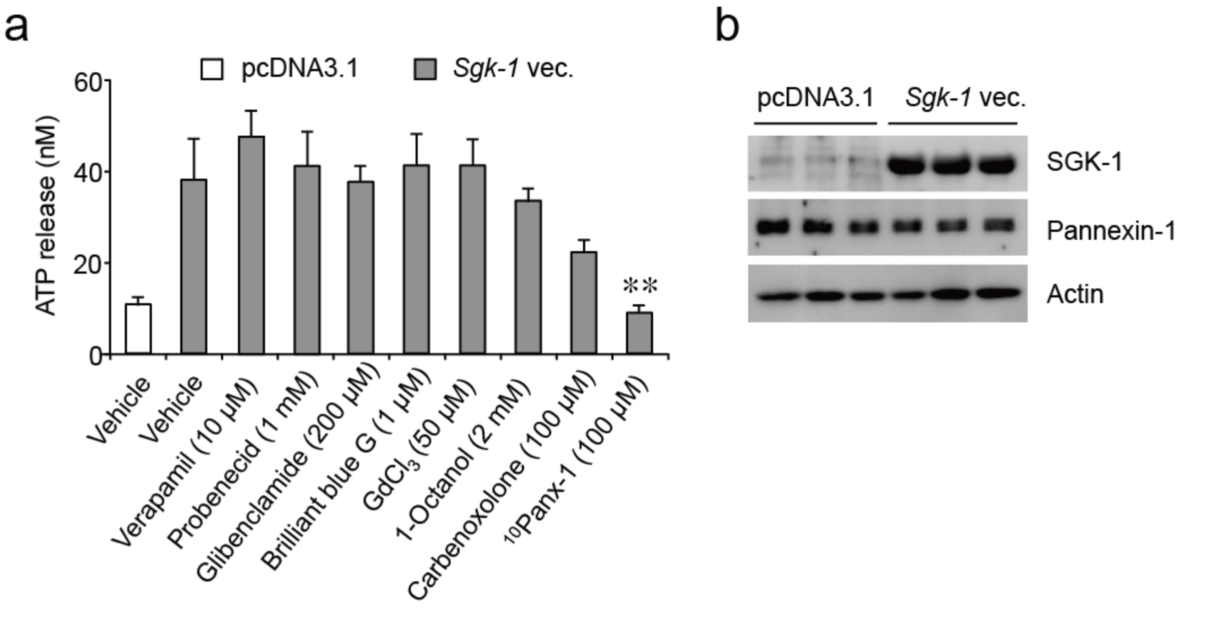

Supplementary Figure 9 SGK-1 enhances astrocytic ATP release via pannexin-1 hemichannels. (**a**), Effects of inhibitors on candidate pathways for the SGK-1-enhanced release of ATP from astrocytes (means ± s.e.m.; *n*=4-6). Cells were transfected with 500 ng of *Sgk-1*-expressing vectors or empty vectors (pcDNA3.1). 24-h post-transfection, cells were treated with inhibitors for 4-h. $^{**}P<0.01$ significant difference from *Sgk-1*-expressing vectors-transfected cells ($F_{9,45}=53.094$, $P=0.005$, ANOVA with Tukey-Kramer post hoc tests). (**b**), Negligible effects of SGK-1 on the expression of pannexin-1 in the membrane fraction of astrocytes. Protein levels were assessed 24-h after transfection. Cells were transfected with 500 ng of *Sgk-1*-expressing vectors (*Sgk-1* vec.) or empty (pcDNA3.1) vectors. Full size images are presented in Supplementary Figure 17.

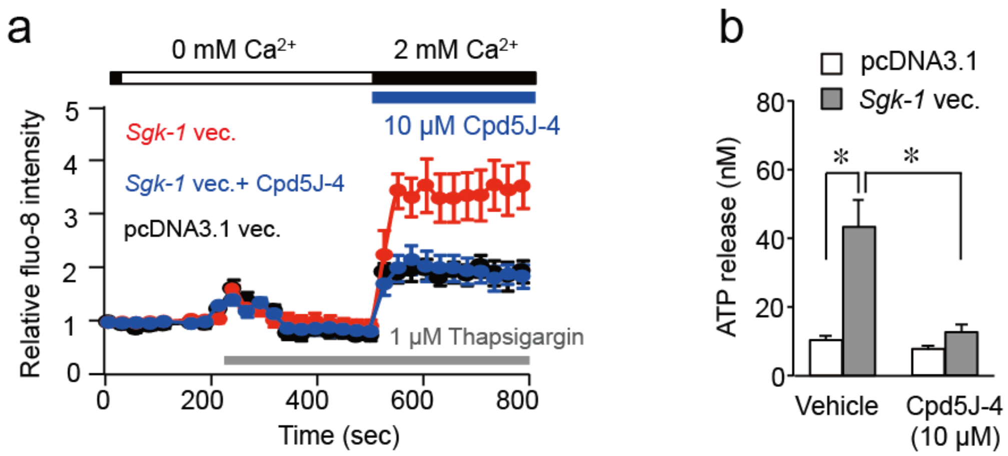

Supplementary Figure 10 SGK-1 enhances store-operated calcium entry in astrocytes via Orai1. (**a**), Tracing of the fluo-8 fluorescence intensity during and after Ca^{2+} depletion in astrocytes transfected with *Sgk-1*-expressing vectors (500 ng) or empty vectors (pcDNA3.1). 24-h post-transfection. Fluo-8 fluorescence was measured in the presence or absence of 10 μM Cpd5J-4 (means ± s.e.m.; *n*=8). Average fluorescence before measurement was set to 1. (**b**), Effects of Cpd5J-4 on the SGK-1-enhanced release of ATP from astrocytes (means ± s.e.m.; *n*=4). *$P < 0.05$ significant difference between two groups ($F_{3,12}$=16.292, P=0.008, ANOVA with Tukey-Kramer PHT).

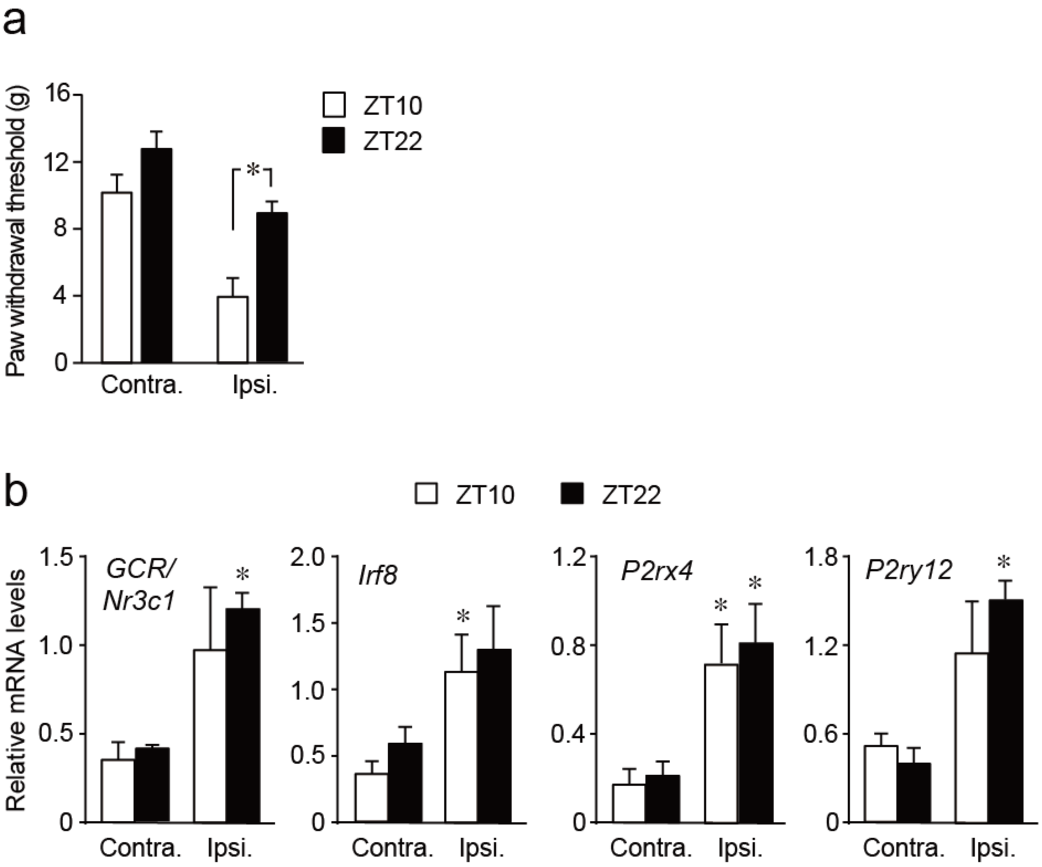

Supplementary Figure 11 Molecular alterations in the spinal cords of PSL rats. Male Sprague Dawley rats were followed partial sciatic nerve ligation (PSL). The pain threshold and mRNA expression were assessed on day 7 after nerve injury. (**a**), Temporal profiles of withdrawal threshold of ipsilateral (ipsi.) and contralateral (contra.) hindpaw of PSL rats (means ± s.e.m.; *n*=3-4). **P*<0.05 significantly different between the two time points ($F_{3,10}$=8.417, *P*=0.004; ANOVA with Tukey-Kramer PHT). (**b**), Temporal mRNA expression profiles of *GCR/Nr3c1*, *Irf8*, *P2rx4,* and *P2ry12* in the spinal cords of PSL rats (means ± s.e.m.; *n*=3-4 per group). **P*<0.05 significantly different from contralateral sides at the corresponding time ($F_{3,10}$=3.679, *P*=0.051 for *GCR/Nr3c1*; $F_{3,10}$=3.517, *P*=0.057 for *Irf8*; $F_{3,10}$=5.771, *P*=0.015 for *P2rx4*; $F_{3,10}$=4.884, *P*=0.024 for *P2ry12*; ANOVA with Tukey-Kramer PHT).

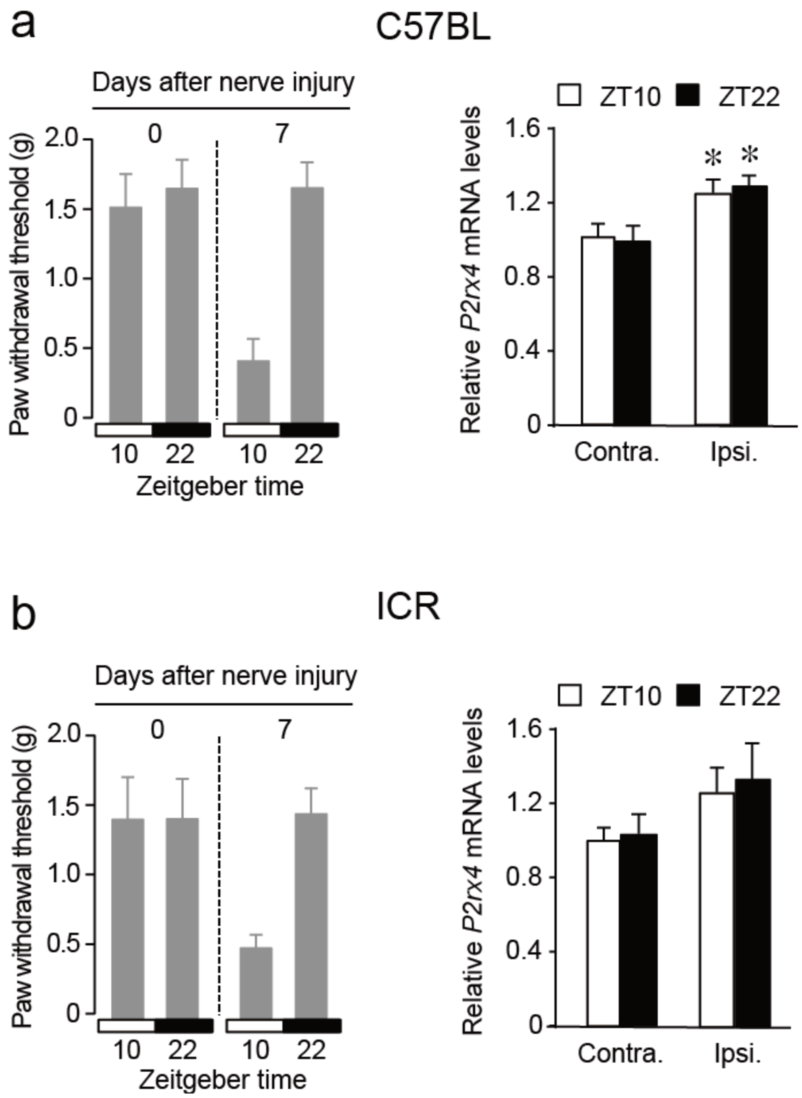

Supplementary Figure 12 Strain-dependent difference in spinal expression of *P2rx4* mRNA in spinal nerve-injured (SNI) mice. Male C57BL and ICR mice were followed L4 spinal nerve transection (**a**), Left panel shows the temporal profiles of the paw withdrawal threshold in SNI C57BL mice (means ± s.e.m.; n=6). A significant time-dependent variation is detected in the paw withdrawal threshold on day 7 after nerve injury ($F_{3,20}$=9.696, P<0.001; ANOVA). Right panel shows the temporal profiles of *P2rx4* mRNA in the spinal cords of SNI C57BL mice on day 7 after nerve injury (means ± s.e.m.; n=7-8). One-way ANOVA with Tukey-Kramer PHT ($F_{3,26}$=6.251, P=0.002) shows that the mRNA levels of *P2rx4* are significantly elevated in the ipsilateral spinal cords (*P<0.05), whereas no significant different is observed in the spinal expression of mRNA between the two time points. (**b**), Left panel shows the temporal profiles of the paw withdrawal threshold in SNI ICR mice (means ± s.e.m.; n=6). A significant time-dependent variation is detected in the paw withdrawal threshold on day 7 after nerve injury ($F_{3,20}$=4.432, P=0.015; ANOVA). Right panel shows the temporal profiles of *P2rx4* mRNA in the spinal cords of SNI ICR mice on day 7 after nerve injury (means ± s.e.m.; n=7-8). One-way ANOVA with Tukey-Kramer post hoc tests showed no significant difference in the *P2rx4* mRNA levels among the groups ($F_{3,26}$=1.536, P=0.229), but the mRNA levels in the ipsilateral sites tended to increase at both time points.

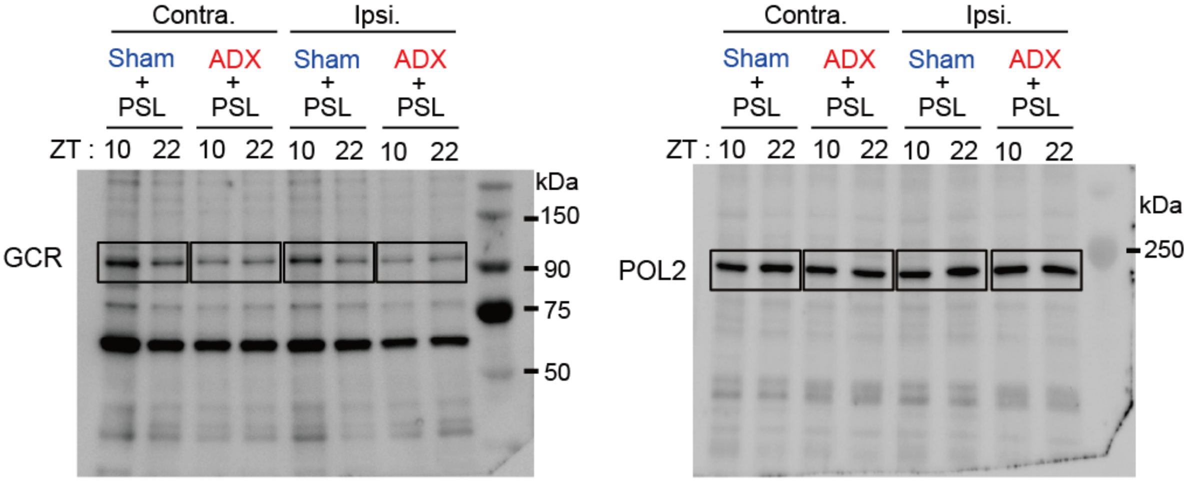

Supplementary Figure 13 Unedited full blots of Figure 4c

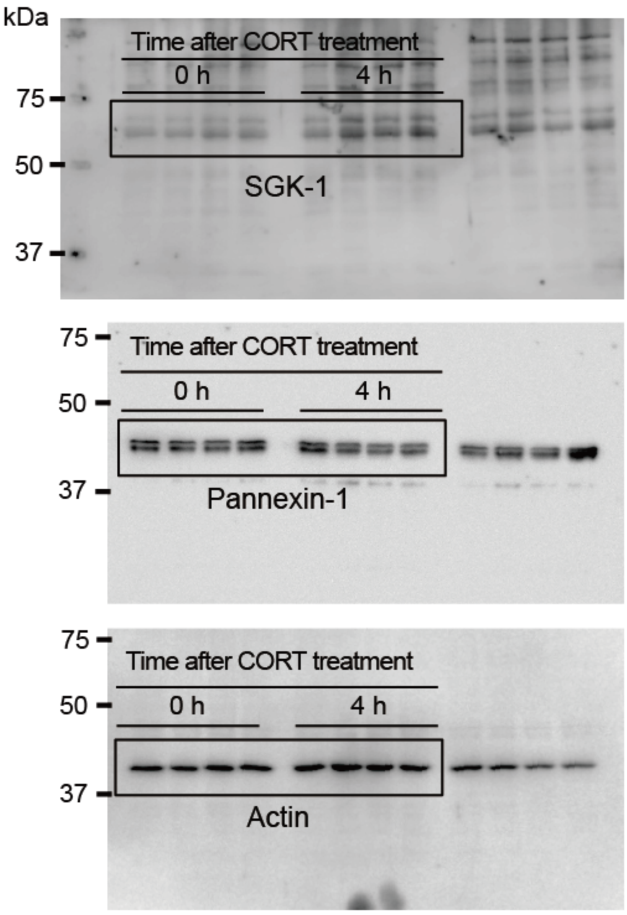

Supplementary Figure 14 Unedited full blots of Figure 6a

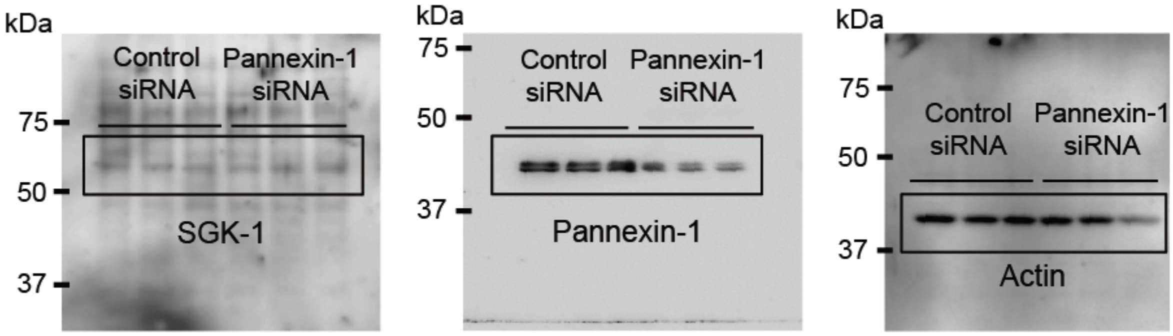

Supplementary Figure 15 Unedited full blots of Figure 6b

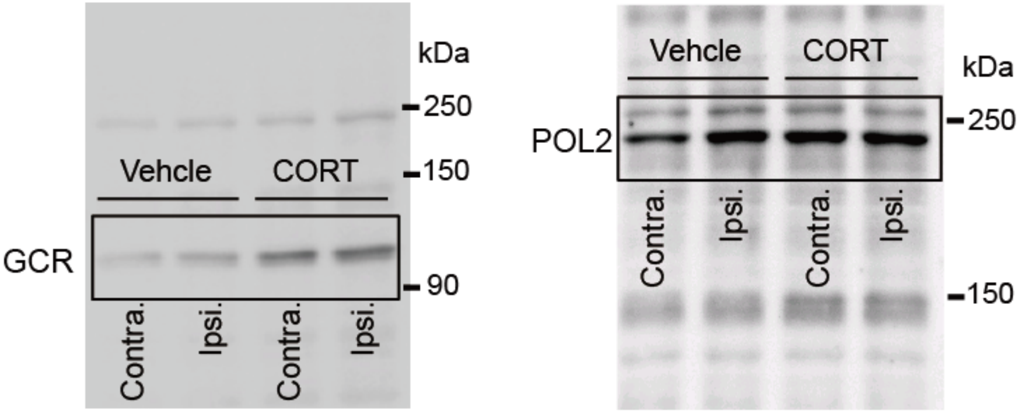

Supplementary Figure 16 Unedited full blots of Supplementary Figure 7b

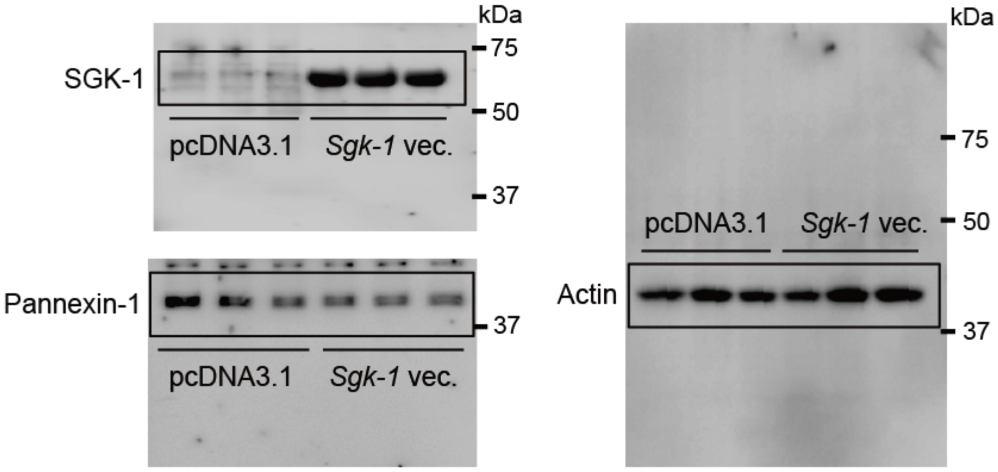

Supplementary Figure 17 Unedited full blots of Supplementary Figure 9b

Supplementary Table 1. Primer sequences for used for quantitative PCR analysis

Mouse *Irf8*	Forward primer<br>Reverse primer	5'- GGATATGCCGCCTATGACACA -3'<br>5'- CATCCGGCCCATACAACTTAG -3'
Mouse *GCR/Nc3r1*	Forward primer<br>Reverse primer	5'- ATGCCGCTATCGAAAATGTC -3'<br>5'- TTAGCGTTTTCAGAAGTGTCT -3'
Mouse *P2rx4*	Forward primer<br>Reverse primer	5'- ACAACGTGTCTCCTGGCTACAAT -3'<br>5'- GTCAAACTTGCCAGCCTTTCC -3'
Mouse *P2rx7*	Forward primer<br>Reverse primer	5'- GCTGCTTGGGAAAAGTCTG -3'<br>5'- TGAGTCGTGGAGAGATAGGGA -3'
Mouse *P2ry6*	Forward primer<br>Reverse primer	5'- TGCTGCTTGGGTAGTGTGTGG -3'<br>5'- GTAAGGCTATGAAGGGCAGC -3'
Mouse *P2ry12*	Forward primer<br>Reverse primer	5'- CACAGAGGGCTTTGG GAACTTA-3'<br>5'- TGGTCCTGCTTCTGCTGAATC -3'
Mouse *Sgk-1*	Forward primer<br>Reverse primer	5'- CGCCAAGTCCCTCTCAACAA -3'<br>5'- TGCCCTTTCCGATCACTTTC -3'
Mouse *18s*	Forward primer<br>Reverse primer	5'- CGGCTACCACATCCAAGGAA -3'<br>5'- GCTGGAATTACCGCGGCT -3'
Mouse *β-Actin*	Forward primer<br>Reverse primer	5'- CCTTCAACACCCCAGCCATG -3'<br>5'- TAGGGCGACATAGCACAGCT -3'
Rat *GCR/Nc3r1*	Forward primer<br>Reverse primer	5'- ATGCCGCTATCGGAAATGTC -3'<br>5'- TTAGGATTTTCCGAAGTGTCT -3'
Rat *Irf8*	Forward primer<br>Reverse primer	5'- GGGTATACTGCCTATGACGCA -3'<br>5'- CGTCCGGCCCATACAGCTTCG -3'
Rat *P2rx4*	Forward primer<br>Reverse primer	5'- ACAATGTGTCTCCTGGCTACAAT -3'<br>5'- GTCAAACTTCCCAGCCTTTCC -3'
Rat *P2ry12*	Forward primer<br>Reverse primer	5'- TAACCATTGACCGATACCTGAAGA -3'<br>5'- TTCGCACCCAAAAGATTGC -3'
Rat *β-Actin*	Forward primer<br>Reverse primer	5'- CTCTTCCAGCCTTCCTTCCTG-3'<br>5'- GTGGTGGTACATGGGTCCGT-3'

Supplementary Methods

Animals and treatments

Five-week-old both sex ICR mice, male C57BL mice, and male Sprague Dawley rats (Charles River Japan) were housed in groups (from 6 to 10 per cage for mice and from 3 to 4 per cage for rats) in a light-controlled room (lights on from ZT0 to ZT12) at 24 ± 1°C and humidity of 60 ± 10% with food and water *ad libitum*. Transection of L4 spinal nerve of mice was performed as described previously[1]. We also prepared PSL model in rats by the same methods for mice. The surgery was performed under sodium pentobarbital (40 mg kg^{-1}, i.p.) and isoflurane anesthesia.

Measurement of CORT levels in the spinal cord of mice

Spinal slices prepared from lumbar segments L4 to L5 were homogenized with CelLytic™ MT cell lysis reagent (Sigma-Aldrich, St Louis, MO). CORT concentrations were measured using a corticosterone enzyme immunoassay kit (Cayman Chemical, Ann Arbor, MI) validated for mouse spinal samples according to the manufacturer's protocol. The protein concentration in lysates was determined using Lowry's method (DC protein assay; Bio-Rad, Hercules, CA). The amount of CORT contents in the spinal cord was expressed as ng per mg protein.

Preparation of rat PSL model and assessment of mechanical allodynia

Five-week-old male male Sprague Dawley (Charles River Japan) were housed in groups of 3-4 per cage in a light-controlled room (lights on from ZT0 to ZT12) at 24 ± 1°C and humidity of 60 ± 10% with food and water *ad libitum*. We prepared partial sciatic nerve ligated rats under sodium pentobarbital (40 mg kg^{-1}, i.p.) and isoflurane anesthesia. The right thigh was shaved and the sciatic nerve was exposed through an incision. Half of the nerve was tightly ligated with 8-0 silk thread and the wound was sutured (Ipsilateral side; right hindpaw). The sciatic nerve of the left hindpaw was also exposed by the same procedure; however, the wound was sutured without nerve ligation (Contralateral side; left hindpaw).

To assess mechanical allodynia, rats were placed individually in an opaque plastic cylinder, which was placed on a wire mesh and habituated for 0.5 h to allow acclimatization to the new environment. Calibrated von Frey filaments (2 - 15 g, North Coast Medical) were then applied to the plantar surfaces of the hind paws of rats. The paw withdrawal threshold was determined using the up-down method[12,13].

Preparation of *Sgk-1*-expressing cells

Primary cultured astrocytes were transfected with *Sgk-1*-expressing vectors or empty (pcDNA3.1) vectors. 24-h post-transfection, cells were treated with various inhibitors or vehicle (ATP assay buffer with 1% DMSO and 1% Ethanol) for 30 min. After this treatment, cells were washed 4 times with ATP assay buffer as described above, and subsequently used for the ATP assay.

Calcium mobilization assay

Astrocytes (2×10^4) were seeded in a 96-well plate. After 24 h, cells were transfected with *Sgk-1*-expressing or empty (pcDNA3.1) vectors. 48 h post-transfection, cells were loaded with 4 μM Fluo-8 AM (Abcam) in 100 μL of Hank's balanced salt solution containing 20 mM HEPES (pH7.4) supplemented with 0.04% Pluronic F-127 and 1% FCS for 30 min at 37°C, followed by further incubation at room temperature for 30 min. Intracellular calcium mobilization was determined by monitoring the fluorescence intensity (excitation at 490 nm and emission at 525 nm) using FlexStation 3 (Molecular Devices, Sunnyvale, CA, USA).

Reference

1. Sorge, R.E., Mapplebeck, J.C., Rosen, S., Beggs, S., Taves, S., Alexander, J.K., Martin, L.J., Austin, J.S., Sotocinal, S.G., Chen, D., Yang, M., Shi, X.Q., Huang, H., Pillon, N.J., Bilan, P.J., Tu, Y., Klip, A., Ji, R.R., Zhang, J., Salter, M.W., & Mogil, J.S. Different immune cells mediate mechanical pain hypersensitivity in male and female mice. *Nat. Neurosci.* **18**,1081–1083 (2015).
